# Selective Oxidation of Vitamin D_3_ Enhanced by Long-Range Effects of a Substrate Channel Mutation in Cytochrome P450_BM3_ (CYP102A1)

**DOI:** 10.1002/chem.202401487

**Published:** 2024-08-22

**Authors:** Wenyu Chen, Jamie N.C. Lynch, Claudia Bustamante, Yuan Zhang, Luet L. Wong

**Affiliations:** 1 Department of Chemistry, University of Oxford, Inorganic Chemistry Laboratory, South Parks Road, Oxford OX1 3QR, UK; 2 Oxford Suzhou Centre for Advanced Research, Ruo Shui Road, Suzhou Industrial Park, Jiangsu, 215123, P.R. China

**Keywords:** vitamin D_3_ oxidation, P450 enzymes, monooxygenases, C–H activation, protein engineering

## Abstract

Vitamin D deficiency affects nearly half the population, with many requiring or opting for supplements with vitamin D_3_ (VD_3_), the precursor of vitamin D (1α,25-dihydroxyVD_3_). 25-HydroxyVD_3_, the circulating form of vitamin D, is a more effective supplement than VD_3_ but its synthesis is complex. We report here the engineering of cytochrome P450_BM3_ (CYP102A1) for the selective oxidation of VD_3_ to 25-hydroxyVD_3_. Long-range effects of the substrate-channel mutation Glu435Ile promoted binding of the VD_3_ side chain close to the heme, enhancing VD_3_ oxidation activity that reached 6.62 g of 25-hydroxyVD_3_ isolated from a 1-litre scale reaction (69.1% yield; space-time-yield 331 mg/L/h).

## Introduction

**Figure 1:**
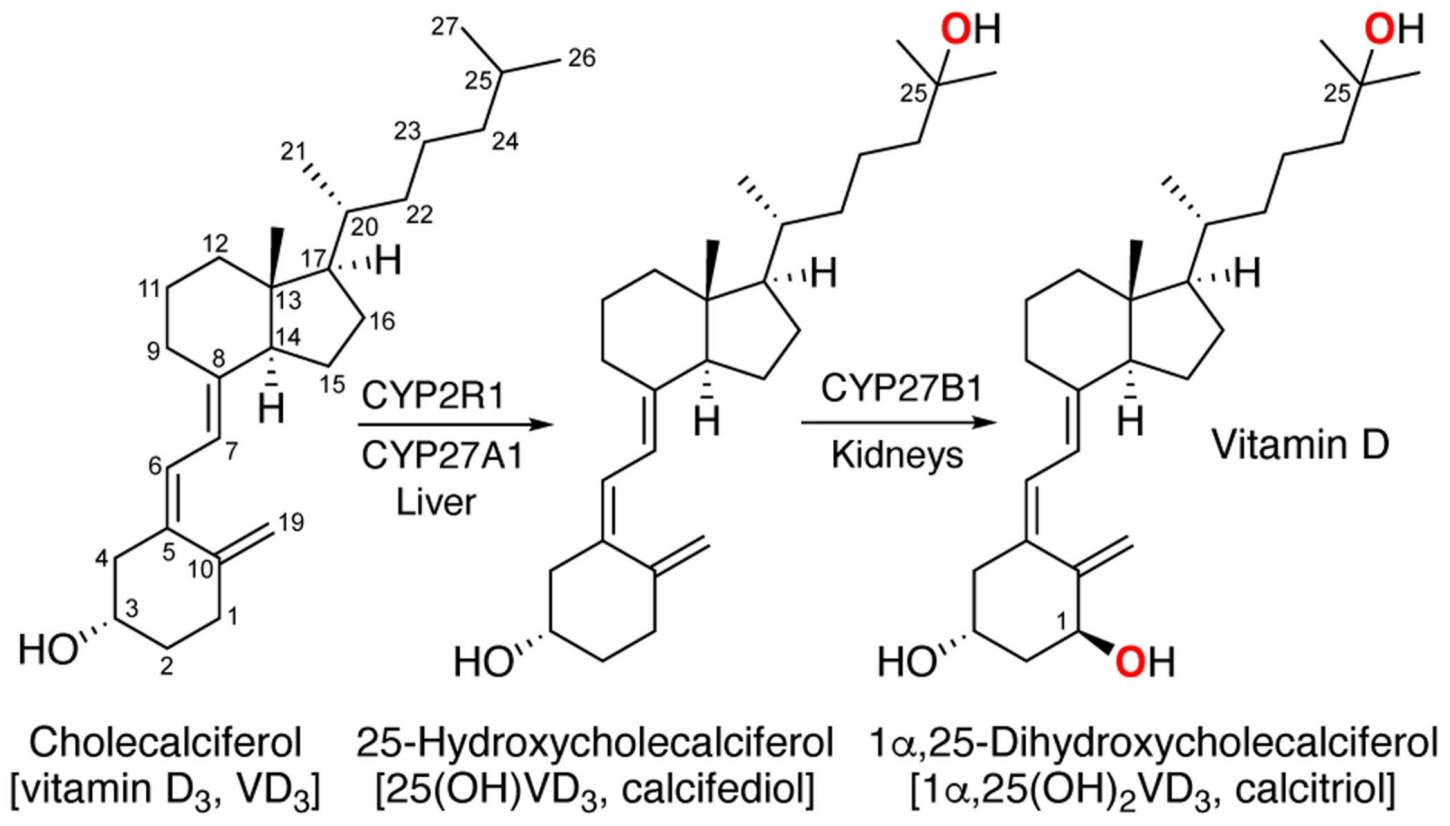
Biosynthetic pathway of vitamin D via sequential hydroxylation of VD_3_.

The vitamin D family of calciferols are secosteroids essential for calcium homeostasis. Whilst best known for its importance in the health of bones, vitamin D plays vital roles in numerous cellular pathways via activation of vitamin D receptors.^[1]^ Vitamin D_3_ (cholecalciferol, VD_3_), the physiological precursor to vitamin D, can be obtained through dietary intake and is biosynthesised in human skin on exposure to UV light via photochemical ring opening of 7-dehydrocholesterol. Vitamin D_2_ (ergocalciferol, VD_2_) is biosynthesised from ergosterol in fungi. The active form of vitamin D is the dihydroxylated derivative 1α,25(OH)_2_VD_3_ formed by sequential oxidation of VD_3_ catalysed by cytochrome P450 (CYP) enzymes. VD_3_ is converted firstly by CYP2R1 and CYP27A1 in the liver to 25-hydroxyVD_3_ [25(OH)VD_3_], the circulating form of vitamin D, and then by CYP27B1 in the kidneys to 1α,25(OH)_2_VD_3_ (Figure 1).^[1b]^ The serum concentration of 25(OH)VD_3_ is the diagnostic marker for vitamin D deficiency which is associated with increased risk of bone fracture as well as conditions such as osteoporosis, cardiovascular disease, and immunodeficiency.^[2]^ A significant proportion of the population are vitamin D deficient and require VD_3_ supplementation. VD_3_ is also an important component in feeds for poultry and farm animals.

25(OH)VD_3_ is a more effective human health supplement than VD_3_ as it is more readily absorbed and more potent in raising serum levels.^[3]^ Supplementation with 25(OH)VD_3_ also has positive effects in the treatment of diseases including hyperglycaemia, Crohn’s disease and chronic kidney and liver conditions.^[4]^ 25(OH)VD_3_ is an approved poultry and animal feed, being more effective than VD_3_ for improving the skeletal health of chicken,^[5]^ the quality and quantity of egg production by hens,^[6]^ and the number and health of progenies from breeding swines.^[7]^ However, while VD_3_ is readily prepared from cholesterol, 25(OH)VD_3_ is synthesised by a complex process from 5,7,24-cholestatrienol produced by a mutant yeast strain via epoxidation–reduction to 25-hydroxy-7-dehydrocholesterol which is converted to 25(OH)VD_3_ in a photochemical process that generates many side products. Selective biological oxidation of VD_3_ to 25(OH)VD_3_ is an attractive alternative for synthesising this important compound in human and animal health.

The mammalian CYP27 and CYP2 families of VD_3_ 25-hydroxylases are membrane-bound enzymes. Their activities and heterologous expression levels are too low for synthetic applications.^[8]^ Since Sasaki *et al*. first reported microbial strains capable of converting VD_3_ to 25(OH)VD_3_,^[9]^ screening programmes have identified more VD_3_-oxidising strains;^[10]^ for example, *Bacillus cereus* zju 4-2 produced 830 mg/L of 25(OH)VD_3_ with a space-time-yield (STY) of 17 mg/L/hour.^[10c]^ CYP105A2 from *Pseudonocardia autotrophica*^[11]^ and CYP105A1 from *Streptomyces griseolus*^[12]^ were the first microbial VD_3_ 25-hydroxylases to be identified. Expression of CYP105A1 in *S. lividans* formed 8 mg/L of 25(OH)VD_3_ from VD_3_ oxidation.^[13]^ Expression of CYP107BR1, another VD_3_ hydroxylase from *P. autotrophica*,^[14]^ in *Rhodococcus erythropolis* and treatment with nisin to facilitate VD_3_ uptake gave 573 mg/L of 25(OH)VD_3_.^[15]^ CYP109A2 and CYP109E1 are VD_3_ hydroxylases from *Bacillus megaterium*;^[16]^ one CYP109A2 variant expressed in *B. megaterium* produced 283 mg/L of 25(OH)VD_3_ in 48 hours.^[17]^

Apart from P450 enzymes, the oxygen-independent molybdenum enzyme sterol C-25 dehydrogenase catalyses the oxidation of VD_3_ to 25(OH)VD_3_ with >99% selectivity and conversion.^[18]^ Although the oxygen-sensitivity of these enzymes had hampered their synthetic application,^[19]^ a recent breakthrough in their expression in the denitrifying bacterium *Thauera aromatica* under semi-aerobic conditions enabled high-density whole-cell (100 mL, OD_578 nm_ = 200) oxidation of VD_3_ to 25(OH)VD_3_ with a yield of 1.89 g/L in 50 hours at a STY of 37 mg/L/h.^[20]^ Fungal unspecific peroxygenases (UPO) have been studied extensively since they only require hydrogen peroxide for catalytic C–H bond oxidation via the same ferryl intermediate as for P450 enzymes.^[21]^ The UPO from *Coprinopsis cinera* catalyses 25-hydroxylation of VD_3_ with >99% conversion and selectivity^[22]^ whereas the *Agrocybe aegerita* enzyme gives 74% of 25(OH)VD_3_, the C-24 and C-26 alcohols and 1α,25(OH)_2_VD_3_.^[23]^ The difficulties in heterologous expression of these glycoproteins are being overcome by protein engineering, and kg-scale processes are being developed.^[24]^ The self-sufficient P450_BM3_ (CYP102A1) from *B. megaterium* is readily expressed to high levels in *Escherichia coli*,^[25]^ and methods for multi-kg scale oxidation have been reported.^[26]^ This enzyme has been the subject of numerous engineering and directed evolution studies for the oxidation of unnatural substrates.^[27]^ Herein we report the application of rational and docking-guided mutagenesis^[28]^ to engineer P450_BM3_ for the selective and scalable C-25 oxidation of both VD_3_ and VD_2_.

## Results and Discussion

A library of 72 P450_BM3_ variants (M1–M72, Table S1, Electronic Supporting Information, ESI) that showed high steroid oxidation activity^[28a]^ was initially screened for VD_3_ oxidation but none showed significant conversion. These variants contained mutations of residues close to the heme, such as F87A, T260G, I263G, A264G and A328G, to create space for steroid binding (Figure S1). We began the design of variants for VD_3_ oxidation with the K19 (H171L/Q307H/N319Y) and R19 (R47L/Y51F/K19) variants as templates. The mutations in these variants are at residues outside of the substrate pocket but they promote the oxidation of unnatural substrates by long-range effects, thus offering excellent starting platforms for substrate pocket mutations to tailor for improved substrate binding and oxidation.^[29]^ The F87A mutation required for steroid oxidation activity was included.^[28a]^ The A82M mutation was added to block the deeper regions of the substrate pocket to constrain VD_3_ to bind near the ferryl oxygen.^[30]^ Glycine substitutions were introduced at T260, A264 and A328 to create space in the vicinity of the heme. The T268S mutation also created space close to the heme while retaining a hydroxyl group to fulfil the role of this residue in oxygen binding and activation. Residues S72, A330 and S332, which were further away from the heme, were substituted with residues with bulkier side chains to promote substrate binding close to the heme. Mutation A184I was included as it was found to increase steroid oxidation activity.^[28a]^

**Figure 2:**
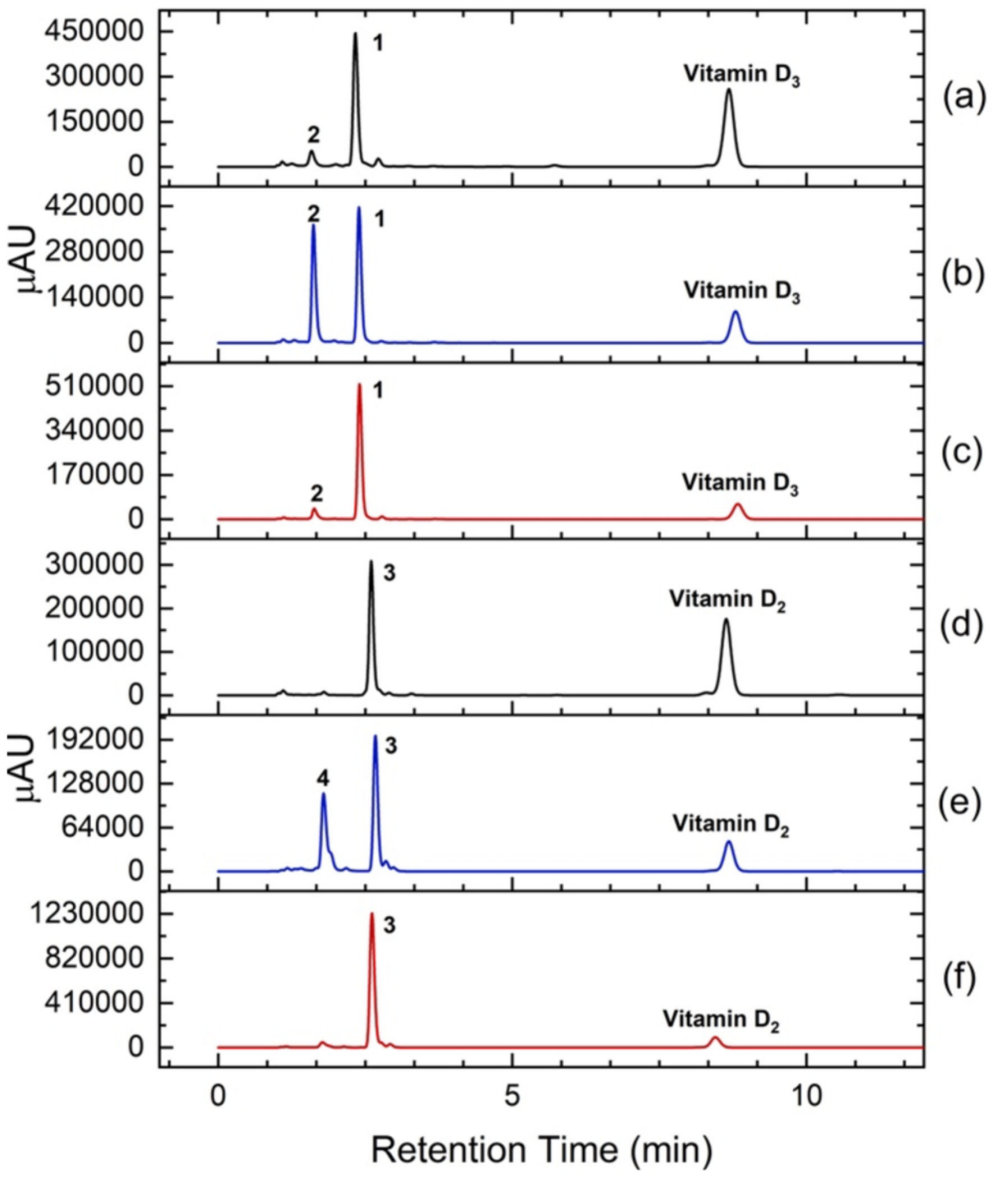
HPLC analysis (C18 column) of the oxidation of vitamin D_3_ and D_2_ by engineered variants of P450_BM3_ showing the formation of 25-hydroxyVD_3_ (**1**), 23,25-dihydroxyVD_3_ (**2**), 25-hydroxyVD_2_ (**3**), and 24,25-dihydroxyVD_2_ (**4**); (a) and (d): with R19/F87A/A82M/A184I/T260G/A328G/S72W (M90, Tables 1 and S5), (b) and (e): with F87V/A82M/A184I/T260G/S72A/E435I (M198, Tables 2 and S5), (c) and (f): with F87I/A82M/A184I/T260G/S72A/E435I (M173, Tables 2 and S5).

A panel of 18 P450_BM3_ variants (M73–M90, Table S2) was generated and screened for VD_3_ oxidation *in vitro*. HPLC analysis of organic extracts from screening scale (0.5 mL, 2 mM substrate, 2 μM P450_BM3_ enzyme) reactions showed that 11 variants possessed >10% VD_3_ conversion activity (Table 1, Figure 2). Two products were purified by silica gel column chromatography from preparative scale reactions and characterised as 25(OH)VD_3_ (**1**) and 23,25-dihydroxyVD_3_ (**2**). Other products were observed but they were not formed in sufficient quantities to be characterised. Mutations A82M, F87A, A184I, T260G, T268S and A328G promoted 25-hydroxylation of VD_3_ to give **1** with up to 74% selectivity (M74, Table 1). All active variants contained the T260G or T268S mutation, and the A82M and A184I mutations were also beneficial. Mutations of A330 and S332 to residues with bulkier side chains decreased activity (M86–M88) but a similar substitution, S72W, led to the highest conversion (54%) and turnover number for C25 oxidation (TON = 375) from this library of enzymes (M90, Table 1, Figure 2). However, further combinations of these activity-enhancing mutations did not increase VD_3_ oxidation activity. We inferred that the mechanisms by which the mutations exerted their effects might be in conflict, leading to lower rather than higher activities when these mutations were combined. Such optimisation plateaus are common in enzyme engineering as the system is trapped in a local maximum. Further improvements often require reverting to the starting point and the use of different combination of mutations.^[31]^

To explore potential conflicts between the effects of mutations in the panel of active variants, we reverted to the F87A variant as the starting point and introduced the apparently effective mutations sequentially to this template variant. As shown in Tables 2 and S4, the activity of the F87A variant was barely detectable but inclusion of the A82M mutation led to 28% VD_3_ conversion and 72% selectivity for **1** (TON = 200, M92, Table 2). The A184I and T260G mutations decreased both conversion and selectivity (M93 and M94) but when introduced together in the F87A/A82M/A184I/T260G variant (M96), they increased VD_3_ conversion to 40% with 75% selectivity for **1**. On the other hand, addition of the S72W, T268S and A328G mutations, which were beneficial to the activity of the R19-based variants, or the mutations R47L, Y51F, H171L, Q307H and N319Y in the R19 base variant, diminished or abolished VD_3_ oxidation activity (data not shown). Although the activities of variants M80 and M90 (Table 1) showed that the detrimental effects of the constituent mutations in the R19 base variant could be rescued when all the mutations were combined, we concluded that further increases in VD_3_ oxidation activity likely required different mutations.

**Figure 3:**
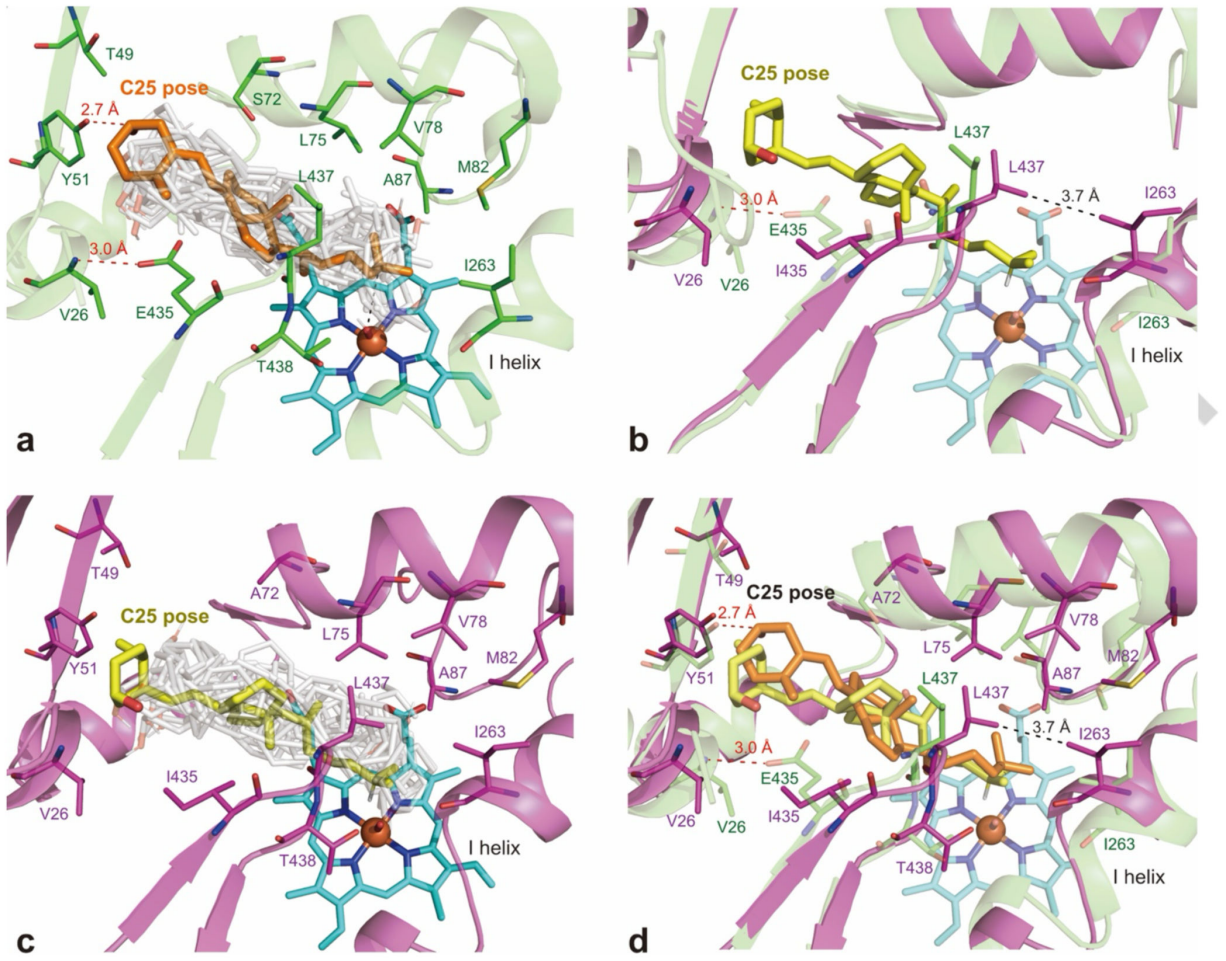
Docking of VD_3_ into molecular dynamics simulation structures of P450_BM3_ variants. (a) The lower energy pose (orange) of the two C-25 poses in the F87A/A82M variant (M92, green) showing the hydrogen bond between the substrate C-3 alcohol and the phenol side chain of Y51 and the pocket high above the heme (cyan) near L75 and L437 where some non-productive (NP) poses (grey) are bound. (b) The lowest energy pose (yellow) of six C-25 poses of VD_3_ docked into the F87A/A82M/A184I/T260G/S72A/E435I variant (M113, magenta) showing the loss of the V26–E435 hydrogen bond and the large movement of the L437 side chain into contact with the side chain of I263. (c) Overlay of the C-25 pose with NP poses (grey) in the F87A/A82M/A184I/T260G/S72A/E435I variant (M113) showing the blocking of the NP pose pocket high above the heme found in the F87A/A82M variant (M90). (d) Overlay of C-25 poses in the two variants highlighting the movement of the L437 side chain into contact with a terminal methyl group of the VD_3_ side chain (yellow) in the F87A/A82M/A184I/T260G/S72A/E435I variant (M113).

To gain an understanding of the role of mutations and to design new mutations to increase activity, VD_3_ was computationally docked into the molecular dynamics (MD) simulation structure of the F87A/A82M variant in its ferryl state. Docking into 12 clustered structures from the four replica simulations led to 108 binding poses which were scattered within the substrate access channel and above the heme. There were 52 productive poses with a carbon centre of the substrate within 4 Å of the ferryl oxygen. Only two poses indicated C-25 oxidation, consistent with the low activity of this variant. These two poses were in similar positions; the C-3 alcohol formed a hydrogen bond with the side chain of Y51 and from there, the VD_3_ molecule extended into the substrate access channel and the space above the heme (Figure 3-A). The A-ring contacted V26, L29, L188, E435 and L437 while the C- and D-rings were in contact with S72, A74, A328, A330, S332, M354, L437 and T438, and there was van der Waals contact between the isopropyl group of the VD_3_ side chain and the mutated residues A87 and M82. Hence, the A82M mutation fulfilled its designed role of promoting VD_3_ binding close to the heme for oxidation by blocking access to the deeper part of the substrate pocket.

Overlays of these C-25 poses with the non-productive (NP) poses (Figure 3-A) showed that the VD_3_ molecule was also in extended conformations in the NP poses, binding within the substrate channel and extending over the heme. Many NP poses had either the A-ring or the side chain of VD_3_ bound in a pocket above the heme defined by the residues L75, V178, L181, M185, L188, L437 and T438. Suitable substitutions at these residues might block this pocket, decreasing the number of NP poses, thus increasing VD_3_ oxidation activity. Other residues that contacted these NP poses but which were further away from the C-25 poses included V26, L29 and V78. The substrate channel residue E435 was of interest since its side chain carbons contacted the A-ring of VD_3_ while the carboxylate group formed a hydrogen bond with the amide–NH of V26 to link the A helix with the β strand containing L437 and T438. Disruption of this hydrogen bond might impart flexibility to this part of the substrate access channel and promote VD_3_ oxidation.

Substitutions with hydrophobic residues with different side chain volumes (Gly, Ala, Val, Leu, Met, Phe, Trp) were introduced at all the above-mentioned residues in the F87A/A82M variant. Disappointingly, all mutations to block the NP pose pocket lowered the VD_3_ oxidation activity. Mutations V26M and L29M increased activity slightly, but the S72A and E435M mutations were effective. The S72A mutation increased VD_3_ conversion to 46% from 28% for the F87A/A82M precursor (M92 and M102, Table 2); the other mutations introduced at this residue (S72G, S72V, S72M, S72F, S72W) lowered the activity. Interestingly, the F87A/A82M/S72A variant gave 65% of **1** as well as 14% of diol **2** whereas the F87A/A82M precursor variant did not give the diol. The E435M mutation was the only introduced substitution at this residue out of the subset (the others were E435G, E435V, E435L, E435F and E435W) to have a positive effect. The variant F87A/A82M/E435M (M101, Table 2) showed 66% conversion and gave 67% of **1** (TON = 440) with 20% of **2**. Introduction of the S72A and E435M mutations to other variants with different mutation combinations revealed that the S72A, A184I and E435M mutations increased activity but they tended to give more of diol **2** (e.g., M105 and M149). The T260G mutation generally lowered conversion but it retarded the formation of **2**. The results showed that both the A184I and T260G mutations were required for high activity (e.g. the series of variants M149, M106, M152 and M118). The variant F87A/A82M/A184I/T260G/S72A/E435M (M118) showed 79% conversion with 73% selectivity for **1** and a TON of 570 (Table 2).

Substitutions of E435 with the other amino acid residues showed that the E435I mutation was even more effective than E435M, with 83% conversion and 75% selectivity for **1** (TON = 620) for the F87A/A82M/A184I/T260G/S72A/E435I variant (M113, Table 2). The E435T mutation had been reported to increase indigo formation via indole oxidation by the A74G/F87V/L188Q variant of P450_BM3_ but hydrophobic substitutions were less effective.^[32]^ In contrast, the E435T mutation (M104) was less effective than E435I (M113) and E435M (M118) in promoting VD_3_ oxidation. We next explored the effect of side-chain volume at residue 87 of the E435I- and E435M-containing variants. The F87V mutation maintained the VD_3_ conversion rate but increased the proportion of diol **2** to ~40% for both the E435I and E435M variants (M198 & M199, Table 2, Figure 2). On the other hand, the F87I mutation increased the selectivity for **1** to over 80%, mainly by disfavouring the formation of diol **2**, while maintaining conversion at 83%, leading to a TON of ~700 for the formation of **1** (M173 & M177).

Docking of VD_3_ into the MD simulation structure of the variant F87A/A82M/A184I/T260G/S72A/E435I showed six C-25 poses compared to two for the F87A/A82M variant, consistent with the higher activity of this variant. Loss of the hydrogen bond between the amide-NH of V26 and the side chain carboxylate of E435 led to movement of both the N-terminal 3_10_ helix and the A helix to widen the substrate channel near the entrance (Figure 3-b). The 435–438 loop swung towards the I helix and the heme, also widening the substrate channel. The L437 side chain showed the largest movement; one δ methyl moved by 10 Å into van der Waals contact with the side chains of L181 and I263 to form a lower roof over the heme and block part of the NP pocket found in the F87A/A82M variant (Figure 3-c). The long-range effect of the E435I mutation was highlighted by the contact between the L437 side chain and a terminal methyl group of the VD_3_ side chain (Figure 3-d) in this variant whereas the L437 side chain contacted the D-ring of VD_3_ in the C-25 poses in the F87A/A82M variant.

Scalability of VD_3_ oxidation by engineered P450_BM3_ was explored with the variant F87I/A82M/A184I/T260G/S72A/E435I (M173) which showed a high TON for 25(OH)VD_3_ formation in screening scale reactions (0.5 mL, 2 mM substrate, 2 μM enzyme). As the VD_3_ concentration was increased while keeping the enzyme concentration at 2 μM, high conversion (86%) and selectivity for 25(OH)VD_3_ (87%) were maintained at 6 mM of VD_3_ (TON = 2230, Table S6, Entry 3), then decreased to 72% and 77%, respectively, at 10 mM (TON = 2750, Table S6, Entry 7). The reaction was scaled to a volume of 1 L, 10 g of VD_3_ (26 mM), and 5 μM enzyme. After stirring with aeration at ambient temperature for 20 hours, the reaction reached 92% conversion (Figure S2, ESI). After work up and silica gel column chromatography, 6.62 g of 25(OH)VD_3_ was isolated (69.1% yield and a STY of 331 mg/L/h based on VD_3_ converted).

Having established high activity and selectivity for C-25 oxidation of VD_3_, this series of active variants was screened for the oxidation of VD_2_. HPLC analysis showed that the variants possessed slightly higher activity for VD_2_ oxidation than for VD_3_ (Table S5, Figure 2). The two major products were isolated by silica gel column chromatography and characterised as 25(OH)VD_2_ (**3**) and 24,25-dihydroxyVD_2_ (**4**). The activity and selectivity data (Table S5) showed similar trends to VD_3_ oxidation – the S72A and E435M mutations increased activity and promoted the formation of diol **4**, the T260G and E435I mutations disfavoured diol formation whereas the F87V mutation promoted it. As with VD_3_, when these mutations were combined, the variant F87I/A82M/A184I/T260G/S72A/E435I (M173) was also the most active for 25-hydroxylation of VD_2_, showing 88% conversion, 92% selectivity and a TON of 810 for the formation of 25(OH)VD_2_.

## Conclusion

Screening of a designed library of variants followed by docking-guided mutagenesis provided engineered variants of P450_BM3_ with high activity and excellent reaction scalability for the selective 25-hydroxylation of VD_3_ to give 25(OH)VD_3_, providing a biocatalytic route to this important human and animal health supplement. Long-range effects resulting from removal of a hydrogen bond linking two secondary structure elements reshaped the P450_BM3_ substrate access channel and binding pocket, leading to increased activity for the oxidation of unnatural substrates. Deliberate weakening of interactions between secondary structure elements offers an additional tool for engineering this evolvable enzyme for synthetic applications.

## Supplementary Material

Additional references cited within the Supporting Information.^[33–48]^

ESI

## Figures and Tables

**Table 1:** Activity and product selectivity for the oxidation of vitamin D_3_ (VD_3_) catalysed by active cytochrome P450_BM3_ variants in the screening library. The substrate-to-enzyme concentration ratio was 1000:1 (2 mM VD_3_, 2 μM P450_BM3_ enzyme). Conversion is the percentage of VD_3_ converted to products. TON is the turnover number of the variant for the formation of 25(OH)VD_3_ (**1**). K19 = H171L/Q307H/N319Y. R19 = R47L/Y51F/K19.

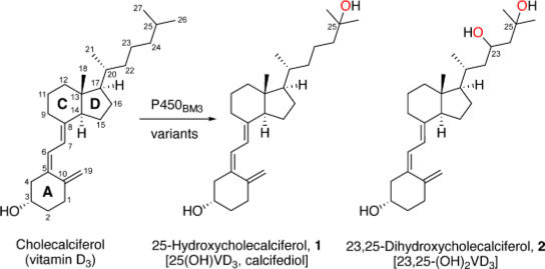						
Variant	Mutations	1	2	Other	Conversion	TON
**M73**	R19 F87A T268S	72%	9%	19%	39%	285
**M74**	R19 F87A T268S A328I	74%	3%	23%	36%	270
**M78**	K19 F87A A82M T260G T268S	50%	3%	47%	18%	90
**M79**	R19 F87A A82M T260G A328G	70%	3%	27%	37%	265
**M80**	R19 F87A A82M A184I T260G	64%	3%	33%	32%	205
**M85**	R19 F87A A82M A184I T260G A328G	64%	8%	28%	30%	195
**M87**	R19 F87A A82M A184I T260G A328G A330V	61%	8%	31%	25%	155
**M86**	R19 F87A A82M A184I T260G A328G A330I	56%	9%	35%	30%	170
**M88**	R19 F87A A82M A184I T260G A328G A330L	52%	10%	38%	29%	150
**M89**	R19 F87A A82M A184I T260G A328G S72F	43%	6%	51%	19%	80
**M90**	R19 F87A A82M A184I T260G A328G S72W	69%	11%	20%	54%	375

**Table 2:** Activity and product selectivity for the oxidation of VD_3_ catalysed by second generation P450_BM3_ variants. The substrate-to-enzyme concentration ratio was 1000:1 (2 mM VD_3_, 2 μM P450_BM3_ enzyme). Conversion is the percentage of substrate converted to products. TON is the turnover number of the variant for the formation of 25(OH)VD_3_ (**1**). –: No detectable activity.

Variant	Mutations	1	2	Other	Conversion	TON
**WT**	—	–	–		–	–
**M91**	F87A	–	–		<3%	–
**M92**	F87A A82M	72%	–	28%	28%	200
**M93**	F87A A82M A184I	30%	–	70%	26%	85
**M94**	F87A A82M T260G	38%	5%	57%	15%	60
**M96**	F87A A82M A184I T260G	75%	5%	20%	40%	300
**M102**	F87A A82M S72A	65%	14%	21%	46%	300
**M105**	F87A A82M A184I S72A	50%	32%	18%	52%	260
**M151**	F87A A82M T260G S72A	47%	4%	49%	21%	100
**M110**	F87A A82M A184I T260G S72A	63%	6%	31%	37%	235
**M101**	F87A A82M E435M	67%	20%	13%	66%	440
**M95**	F87A A82M A184I E435M	61%	21%	18%	53%	320
**M153**	F87A A82M T260G E435M	73%	6%	21%	52%	380
**M97**	F87A A82M A184I T260G E435M	72%	5%	23%	50%	360
**M149**	F87A A82M S72A E435M	45%	41%	14%	55%	245
**M106**	F87A A82M A184I S72A E435M	71%	11%	18%	54%	390
**M152**	F87A A82M T260G S72A E435M	64%	10%	26%	42%	265
**M118**	F87A A82M A184I T260G S72A E435M	73%	15%	12%	79%	570
**M113**	F87A A82M A184I T260G S72A E435I	75%	14%	11%	83%	620
**M104**	F87A A82M A184I T260G S72A E435T	70%	6%	24%	77%	540
**M199**	F87V A82M A184I T260G S72A E435M	49%	40%	11%	77%	380
**M198**	F87V A82M A184I T260G S72A E435I	50%	42%	8%	83%	410
**M173**	F87I A82M A184I T260G S72A E435I	83%	8%	9%	83%	690
**M177**	F87I A82M A184I T260G S72A E435M	81%	10%	9%	83%	670
