# Supplementary material for: Selective Oxidation of Vitamin D3 Enhanced by Long-Range Effects of a Substrate Channel Mutation in Cytochrome P450BM3 (CYP102A1)
Source: Chemistry. Author manuscript; Available in PMC 2025 Jun 19. (PMC7617770; doi:10.1002/chem.202401487)
Supplement: ESI [file EMS206374-supplement-ESI.pdf]

Supporting Information

S1. Experimental Procedures

S1.1 General

General reagents, chemicals and HPLC grade solvents were purchased from Alfa-Aesar, Fisher Scientific, Fluorochem and Sigma-Aldrich, UK. Vitamins D_2 and D_3 were from Enzo Life Sciences and Merck Life Science, UK. Media components, kanamycin and isopropyl-β-D-thiogalactopyranoside (IPTG) were supplied by Melford Laboratories, UK. Hen egg white lysozyme was from Sigma-Aldrich. $NADP^+$ monosodium salt was from Prozomix, UK, and glucose dehydrogenase (GDH) was from Codexis, California, USA. Oligonucleotides were supplied by Eurofins Genetic Service, UK. Vitamin D oxidation products were purified by flash silica gel column chromatography using Geduran Silica 60, 40–63 μm (Fisher Scientific, UK) and eluting with increasingly polar mixtures of petroleum ether (bp 40–60 °C) and ethyl acetate. Analytical thin-layer chromatography was performed with petroleum ether/ethyl acetate solvent systems and bands were visualised under ultraviolet light. ^{1}H, ^{13}C, COSY, HSQC, HMBC and NOESY NMR spectra were acquired on Bruker AVIII-500 (500/125 MHz), Bruker AVIII-400 (400/100 MHz), or Bruker AV-400 (400/100 MHz) spectrometers. High resolution mass spectra (HRMS) were obtained on a Bruker microTOF spectrometer. UV-vis spectra were acquired on a Varian CARY50 spectrophotometer using 1 cm pathlength quartz cuvettes. Reverse-phase high performance liquid chromatography (HPLC) analyses were carried out with a C18 column (5 μm, 100 mm × 4.6 mm, Kinetex, UK) on a Shimadzu Prominence system equipped with a photodiode array detector and an autosampler. The sample injection volume was 10 μL and the column oven was set at 40 °C. Analyses of the oxidation product profiles of vitamins D_2 and D_3 were conducted with a mixture of 95% acetonitrile and 5% water at a flow rate of 1 mL/min for 20 min. Retention times for compounds were, vitamin D_3, 8.393 min; 25-hydroxy-vitamin D_3 (**1**), 2.338 min; 23,25-dihydroxy-vitamin D_3 (**2**),

1.607 min; vitamin D_2, 7.842 min; 25-hydroxy-vitamin D_2 (**3**), 2.695 min; 24,25-dihydroxy-vitamin D_2 (**4**), 1.964 min.

S1.2 Enzymes and molecular biology

Genes encoding CYP102A1 enzymes were cloned in the pET28+ vector by NcoI and BamHI restriction sites.[33] Site-directed mutagenesis was carried out by standard PCR-based protocols using KOD Hot Start DNA Polymerase toolkit from Sigma-Aldrich, UK. The presence of the target mutation(s) was confirmed by DNA sequencing. The relevant plasmid was transformed into chemically competent *E. coli* BL21 (DE3) for enzyme production and subsequent purification as described previously.[34] P450 enzyme concentrations were determined by the $Fe^{II}(CO)$ difference spectrum method of Omura and Sato using $\varepsilon_{450\text{–}490\text{nm}} = 91000\ M^{-1}\ cm^{-1}$.[35]

S1.3 Activity screening and preparative scale reactions

The vitamin D_2 and D_3 substrates were dissolved in methanol and added as a stock solution at 200 mM concentration. Enzymatic activity screening was carried out in 24-well plates. The 0.5 mL reaction mixture in each well (200 mM phosphate buffer, pH 7.9) contained 2 mM vitamin D substrate, 2 µM CYP102A1 variant, 4 U/mL GDH (4 U/µL stock) and 100 mM glucose (2 M stock). $NADP^+$ monosodium salt (40 µM, 40 mM stock) was added to initiate the reaction. Plates were shaken at 20 °C for 16 h at 120 rpm. Each reaction was then extracted with 0.3 mL ethyl acetate. After centrifugation at 14300 ×*g* to separate the phases, the organic extracts were analysed by HPLC.

Preparative scale reactions (50–1000 mL) with selected enzymes for the characterisation of vitamin D metabolites were carried out for 6–16 h under the same conditions as screening scale reactions. Progress of the reaction was monitored by analysis of organic extracts of 0.5 mL aliquots. The reaction mixture was then thrice extracted, each time with an equal volume of ethyl acetate. The combined organic extracts were washed with water and brine, dried with $Na_2(SO_4)$ and the solvent was removed by rotary evaporation. Products were purified by silica gel column chromatography.

S1.4 Molecular dynamics (MD) simulations and substrate docking

MD simulations were performed on the heme domain of CYP102A1 variants. Mutations were introduced to the crystal structure of the heme domain of the wild type enzyme with *N*-palmitoylglycine (NPG) bound within the active site (PDB code: 1jpz)[36] using Pymol. The bound molecule of NPG was removed. MD simulations were carried out within the GROMACS 2018.6 suite.[37] The protein was prepared using the Amber 99SB*-ILDN force field[38] with TIP3P water.[39] The heme was simulated in the compound I state, using parameters from Shahrokh, *et al*.[40] The protein was placed into the centre of an octahedral box, with a minimum distance of 10 nm to any box edge, followed by solvation with approximately 18000 water molecules and charge was neutralised by the addition of Na^+ ions. Steepest-descent energy minimisation was performed until the maximum force was <500 kJ mol^{-1} nm^{-2}. The system was modelled by periodic boundary conditions. Electrostatic interactions were treated by the particle mesh Ewald method[41] while bond lengths involving hydrogen atoms were constrained with the LINCS algorithm.[37b] Short-range non-bonded interactions were calculated with a 1 nm cut-off and a timestep of 2 fs. For equilibration steps, all protein backbone Cα atoms were restrained with a positional restraint force constant of 1000

kJ mol^{-1} nm^{-2}. A NVT equilibration step was performed at 298 K for 100 ps, using the modified Berendsen thermostat.[42] This was followed by an NPT equilibration step for 1250 ps utilizing the Berendsen barostat with a time constant of 1 ps.[43] All positional restraints were then removed, and 100 ns of production MD was performed in quadruplicate using the Parrinello-Rahman barostat with a time constant of 5 ps.[44] Structures were recorded every 10 ps. Stability of the simulations was confirmed by monitoring the RMSD of the backbone Cα atoms before the individual trajectories were clustered using the Daura algorithm with a cut-off of 1.2 Å. The three most populated clusters of each replica, with a population cut-off of 5% of all trajectories, were used as receptor structures for docking studies, which were performed in Autodock Vina. All water molecules were removed from the structure prior to the docking calculation and both receptor and substrate were treated as rigid entities. The docking site was defined as a 30 × 30 × 30 Å box centred on the ferryl oxygen, and poses were ranked using the Autodock Vina scoring function.

S2. Lists of P450$_{BM3}$ (CYP102A1) variants

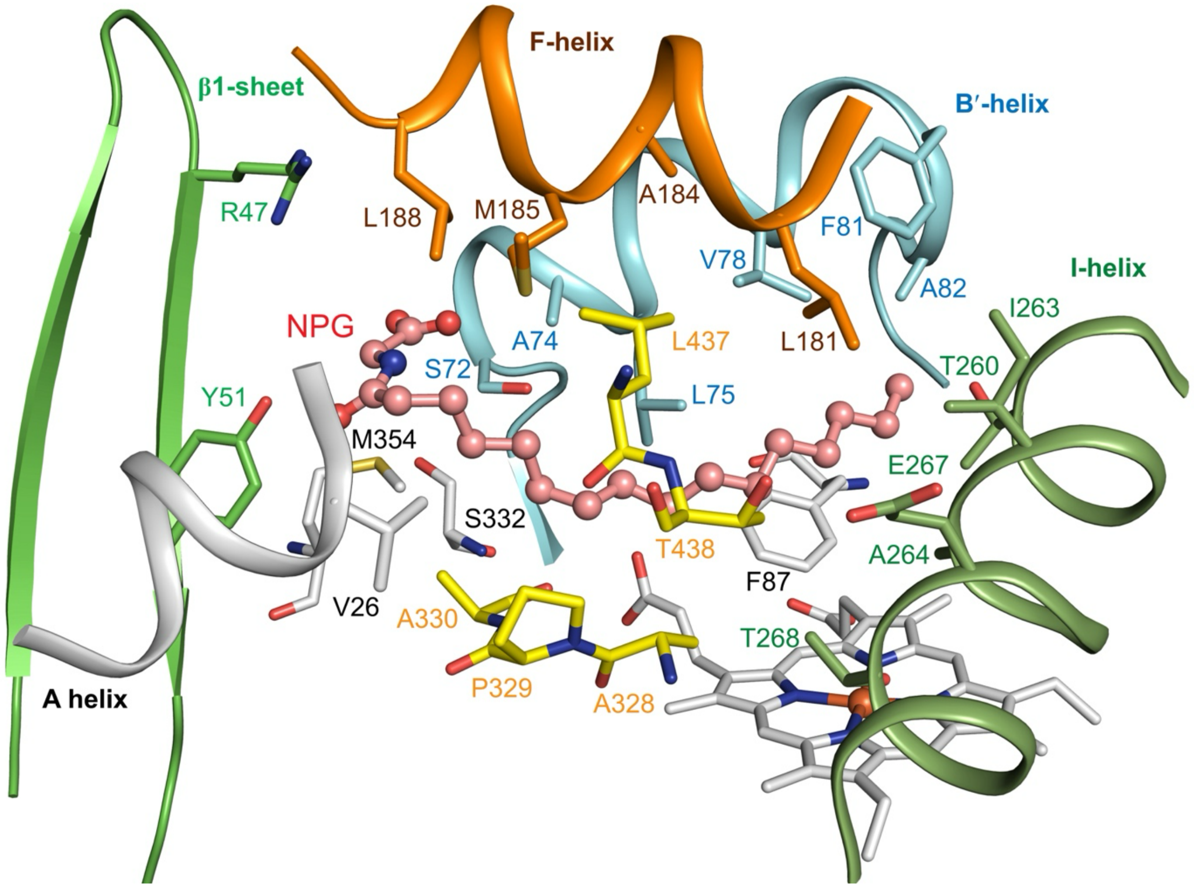

Figure S1. The active site structure of the heme domain of wild-type P450$_{BM3}$ with bound *N*-palmitoylglycine (NPG), highlighting the substrate-binding residues targeted for mutagenesis to generate the screening library of variants (pdb code: 1jpz).[34] Secondary structural elements and residues within them are highlighted by different colours.

Table S1. The initial screening library of $P450_{BM3}$ variants.

GV = A74G/F87V; GVQ = A74G/F87V/L188Q; K19 = H171L/Q307H/N319Y; R19 = R47L/Y51F/H171L/Q307H/N319Y.

Variant	Mutations	Variant	Mutations
**M1**	GV/A184I/A264G	**M37**	R19/F87A/A184I/I259G/I263G
**M2**	GV/A184I/A264G/A328G	**M38**	R19/F87A/A184I/I263G/A264G
**M3**	GV/A184I/A328G	**M39**	R19/F87A/A184I/I263G/A264G/A328G
**M4**	GV/A184I/A328G/T260G	**M40**	R19/F87A/A184I/I263G/A328G
**M5**	GV/A184I/I263G	**M41**	R19/F87A/A184I/I263W
**M6**	GV/A184I/I263G/A264G	**M42**	R19/F87A/A184I/L262G
**M7**	GV/A184I/I263G/A264G/A328G	**M43**	R19/F87A/A184I/S270G
**M8**	GV/A184I/I263G/A328G	**M44**	R19/F87A/A184I/T260G
**M9**	GV/A184I/I263W	**M45**	R19/F87A/A184I/T269G
**M10**	GV/A184I/T260G	**M46**	R19/F87A/A264G
**M11**	GVQ/A264G/P329G/A330P	**M47**	R19/F87A/A328G
**M12**	GVQ/A264G/P329G/A330W	**M48**	R19/F87A/A328G/A264G
**M13**	GVQ/A328G	**M49**	R19/F87A/A328G/I259G/I263G
**M14**	GVQ/I263G/A264G	**M50**	R19/F87A/A328G/I263G/A264G
**M15**	GVQ/I263G/A264G/A328G	**M51**	R19/F87A/A328G/I263W
**M16**	GVQ/T260G	**M52**	R19/F87A/A328G/L262G
**M17**	K19/F87V/A264G	**M53**	R19/F87A/A328G/P329G/A330G
**M18**	K19/F87V/A184I	**M54**	R19/F87A/A328G/P329G/A330G/T260G
**M19**	K19/F87V/I263G	**M55**	R19/F87A/A328G/S270G
**M20**	K19/A82M/F87A/A264G	**M56**	R19/F87A/A328G/T260G
**M21**	K19/A82M/F87A/A264G/A328G	**M57**	R19/F87A/A328G/T269G
**M22**	K19/A82M/F87A/A328G/T260G	**M58**	R19/F87A/F81W/A328G/T260G
**M23**	K19/A82M/F87A/A330W	**M59**	R19/F87A/G265GG
**M24**	K19/A82M/F87A/F261G	**M60**	R19/F87A/I259G/I263G
**M25**	K19/A82M/F87A/I263G/A264G	**M61**	R19/F87A/I263G/A264G
**M26**	K19/A82M/F87A/I263G/A264G/A328G	**M62**	R19/F87A/I263G/A328G
**M27**	K19/A82M/F87A/I263G/A264G/A330W	**M63**	R19/F87A/I263G/P329G/A330P
**M28**	K19/A82M/F87A/I263G/A328G	**M64**	R19/F87A/I263G/P329G/A330W
**M29**	K19/A82M/F87A/I263G/A330W	**M65**	R19/F87A/I263W
**M30**	K19/A82M/F87A/I263W	**M66**	R19/F87A/L262G
**M31**	K19/A82M/F87A/L262G	**M67**	R19/F87A/P329G/A330P
**M32**	K19/A82M/F87A/S270G	**M68**	R19/F87A/P329G/A330W
**M33**	K19/A82M/F87A/T260G	**M69**	R19/F87A/P329G/A330W/T260G
**M34**	R19/F87A/A184I/A264G	**M70**	R19/F87A/S270G
**M35**	R19/F87A/A184I/A264G/A328G	**M71**	R19/F87A/T260G
**M36**	R19/F87A/A184I/A328G/T260G	**M72**	R19/F87A/T269G

Table S2. P450$_{BM3}$ variants by rational design.

K19 = H171L/Q307H/N319Y; R19 = R47L/Y51F/H171L/Q307H/N319Y.

Variant	Mutations
**M73**	R19/F87A/T268S
**M74**	R19/F87A/T268S/A328I
**M75**	K19/F87A/A82M/T260G
**M76**	K19/F87A/A82M/A184I/T260G
**M77**	R19/F87A/A82M/T260G
**M78**	K19/F87A/A82M/T260G/T268S
**M79**	R19/F87A/A82M/T260G/A328G
**M80**	R19/F87A/A82M/A184I/T260G
**M81**	R19/F87I/A82M/A184I/T260G
**M82**	R19/F87V/A82M/A184I/T260G
**M83**	R19/F87A/A82M/A184I/A328G
**M84**	R19/F87A/A82M/A184I/T260G/S72L
**M85**	R19/F87A/A82M/A184I/T260G/A328G
**M86**	R19/F87A/A82M/A184I/T260G/A328G/A330I
**M87**	R19/F87A/A82M/A184I/T260G/A328G/A330V
**M88**	R19/F87A/A82M/A184I/T260G/A328G/A330L
**M89**	R19/F87A/A82M/A184I/T260G/A328G/S72F
**M90**	R19/F87A/A82M/A184I/T260G/A328G/S72W

Table S3. Variants designed by docking-guided mutagenesis.

K19 = H171L/Q307H/N319Y; R19 = R47L/Y51F/H171L/Q307H/N319Y.

Variant	Mutations
**M91**	F87A
**M92**	F87A/A82M
**M93**	F87A/A82M/A184I
**M94**	F87A/A82M/T260G
**M95**	F87A/A82M/A184I/E435M
**M96**	F87A/A82M/A184I/T260G
**M97**	F87A/A82M/A184I/T260G/E435M
**M98**	F87A/A82M/A184I/T260G/L150P/M354G/E435M
**M99**	F87A/A82M/A184I/T260G/M354L/E435M
**M100**	F87A/A82M/A184I/T260G/S332A
**M101**	F87A/A82M/E435M
**M102**	F87A/A82M/S72A
**M103**	F87A/A82M/S72A/A184F/T260G/E435M
**M104**	F87A/A82M/S72A/A184I/T260G/E435T
**M105**	F87A/A82M/A184I/S72A
**M106**	F87A/A82M/S72A/A184I/E435M
**M107**	F87A/A82M/S72A/A184I/N239H/T260G/E435M
**M108**	F87A/A82M/S72A/A184I/T260A
**M109**	F87A/A82M/S72A/A184I/T260A/E435M
**M110**	F87A/A82M/S72A/A184I/T260G
**M111**	F87A/A82M/S72A/A184I/T260G/E435D
**M112**	F87A/A82M/S72A/A184I/T260G/E435H
**M113**	F87A/A82M/S72A/A184I/T260G/E435I
**M114**	F87A/A82M/S72A/A184I/T260G/E435I/V26H
**M115**	F87A/A82M/S72A/A184I/T260G/E435I/V26L
**M116**	F87A/A82M/S72A/A184I/T260G/E435I/V26M
**M117**	F87A/A82M/S72A/A184I/T260G/E435L
**M118**	F87A/A82M/S72A/A184I/T260G/E435M
**M119**	F87A/A82M/S72A/A184I/T260G/E435M/N319Y
**M120**	F87A/A82M/S72A/A184I/T260G/E435M/Q307H
**M121**	F87A/A82M/S72A/A184I/T260G/E435M/V26H
**M122**	F87A/A82M/S72A/A184I/T260G/E435M/V26M
**M123**	F87A/A82M/S72A/A184I/T260G/E435N
**M124**	F87A/A82M/S72A/A184I/T260G/E435Q
**M125**	F87A/A82M/S72A/A184I/T260G/E435R
**M126**	F87A/A82M/S72A/A184I/T260G/E435T/V26H
**M127**	F87A/A82M/S72A/A184I/T260G/E435T/V26L
**M128**	F87A/A82M/S72A/A184I/T260G/E435T/V26M
**M129**	F87A/A82M/S72A/A184I/T260G/E435W
**M130**	F87A/A82M/S72A/A184I/T260G/E435Y

Variant	Mutations
**M131**	F87A/A82M/S72A/A184I/T260G/H171L/E435M
**M132**	F87A/A82M/S72A/A184I/T260G/H171L/L181F/E435M
**M133**	F87A/A82M/S72A/A184I/T260G/H171L/N239H/E435M
**M134**	F87A/A82M/S72A/A184I/T260G/H171L/Q403P
**M135**	F87A/A82M/S72A/A184I/T260G/L181F/N239H/E435M
**M136**	F87A/A82M/S72A/A184I/T260G/Q403P/E435M
**M137**	F87A/A82M/S72A/A184I/T260G/V26L/E435M
**M138**	F87A/A82M/S72A/A184I/T260G/V26L/H171L
**M139**	F87A/A82M/S72A/A184I/T260G/V26L/H171L/E435M
**M140**	F87A/A82M/S72A/A184I/T260G/V26L/N239H/E435M
**M141**	F87A/A82M/S72A/A184I/T260G/V26L/Q403P
**M142**	F87A/A82M/S72A/A184I/T260G/V26L/Y51F
**M143**	F87A/A82M/S72A/A184I/T260G/Y51F/H171L
**M144**	F87A/A82M/S72A/A184I/T260G/Y51F/Q403P
**M145**	F87A/A82M/S72A/A184L/T260G/E435I
**M146**	F87A/A82M/S72A/A184M/T260G/E435I
**M147**	F87A/A82M/S72A/A184M/T260G/E435M
**M148**	F87A/A82M/S72A/A184V/T260G/E435I
**M149**	F87A/A82M/S72A/E435M
**M150**	F87A/A82M/S72A/L181F/A184G/T260G
**M151**	F87A/A82M/S72A/T260G
**M152**	F87A/A82M/S72A/T260G/E435M
**M153**	F87A/A82M/T260G/E435M
**M154**	F87A/A82M/V178F/T260G/E435M
**M155**	F87A/A82M/V178W/T260G/E435M
**M156**	F87A/S72A/A184I/T260G/E435M
**M157**	F87A/S72A/A184I/T260G/E435T
**M158**	F87I/A82M
**M159**	F87I/A82M/A184I/E435M
**M160**	F87I/A82M/A184I/T260G
**M161**	F87I/A82M/A184I/T260G/E435I/S72V
**M162**	F87I/A82M/A184I/T260G/E435I/S72W
**M163**	F87I/A82M/A184I/T260G/E435M
**M164**	F87I/A82M/A184I/T260G/E435M/S72V
**M165**	F87I/A82M/A184I/T260G/E435M/S72W
**M166**	F87I/A82M/E435M
**M167**	F87I/A82M/S72A
**M168**	F87I/A82M/S72A/A184I
**M169**	F87I/A82M/S72A/A184I/E435M
**M170**	F87I/A82M/S72A/A184I/N239H/T260G/E435I
**M171**	F87I/A82M/S72A/A184I/N239H/T260G/E435M
**M172**	F87I/A82M/S72A/A184I/T260G
**M173**	F87I/A82M/S72A/A184I/T260G/E435I

Variant	Mutations
**M174**	F87I/A82M/S72A/A184I/T260G/E435I/L75S
**M175**	F87I/A82M/S72A/A184I/T260G/E435I/M185T
**M176**	F87I/A82M/S72A/A184I/T260G/E435I/M354F
**M177**	F87I/A82M/S72A/A184I/T260G/E435M
**M178**	F87I/A82M/S72A/A184I/T260G/E435M/L188S
**M179**	F87I/A82M/S72A/A184I/T260G/E435M/L29A
**M180**	F87I/A82M/S72A/A184I/T260G/E435M/L29M
**M181**	F87I/A82M/S72A/A184I/T260G/E435M/L75S
**M182**	F87I/A82M/S72A/A184I/T260G/E435M/L75T
**M183**	F87I/A82M/S72A/A184I/T260G/E435M/M354F
**M184**	F87I/A82M/S72A/E435M
**M185**	F87I/A82M/S72A/T260G
**M186**	F87I/A82M/S72A/T260G/E435M
**M187**	F87I/A82M/S72A/V178F/T260G/E435I
**M188**	F87I/A82M/S72A/V178F/T260G/E435M
**M189**	F87I/A82M/S72A/V178W/T260G/E435I
**M190**	F87I/A82M/S72A/V178W/T260G/E435M
**M191**	F87I/A82M/T260G
**M192**	F87I/A82M/T260G/E435M
**M193**	F87S/A82M/S72A/A184I/T260G/E435M
**M194**	F87T/A82M/S72A/A184I/T260G/E435I
**M195**	F87T/A82M/S72A/A184I/T260G/E435M
**M196**	F87V/A82M/S72A/A184I/N239H/T260G/E435I
**M197**	F87V/A82M/S72A/A184I/N239H/T260G/E435M
**M198**	F87V/A82M/S72A/A184I/T260G/E435I
**M199**	F87V/A82M/S72A/A184I/T260G/E435M
**M200**	K69I/F87A/A82M/A184I/T260G/E435M
**M201**	K69R/F87A/A82M/A184I/T260G/E435M
**M202**	F87I/A82M/S72A/A184I/M185T/T260G/E435M/L29M
**M203**	L29M/S72A/L75S/A82M/F87T/A184I/T260G/E435I
**M204**	L29M/S72A/V78M/A82M/F87T/A184I/T260G/E435I
**M205**	R19/F87A/A82M/A184I/T260G/E435I
**M206**	R19/F87A/A82M/A184I/T260G/E435M
**M207**	S72A/A82M/F87T/A184I/M185T/T260G/E435M
**M208**	S72A/L75S/A82M/F87T/A184I/T260G/E435I
**M209**	S72A/V78M/A82M/F87T/A184I/T260G/E435I
**M210**	F87A/A82M/S72A/A184I/T260G/E435M/Y51F
**M211**	R19/F87I/A82M/A184I/T260G
**M212**	R19/F87T/A82M/A184I/T260G
**M213**	R19/F87V/A82M/A184I/T260G
**M214**	R19/F87A/A82M/A184I/T260G/E435T
**M215**	R19/F87A/A82M/A184I/T260G/S72A

S3. Vitamin D oxidation activity and selectivity

Table S4. Activity and product selectivity for the oxidation of vitamin D_3 (VD_3) catalysed by $P450_{BM3}$ variants (K19 = H171L/Q307H/N319Y. R19 = R47L/Y51F/H171L/Q307H/N319Y). The substrate-to-enzyme concentration ratio was 1000:1 (2 mM VD_3, 2 μM $P450_{BM3}$ enzyme). Conv. is the percentage of VD_3 converted to products. TON is the turnover number of the variant for the formation of 25(OH)VD_3 (**1**). All percentages are rounded to the nearest integer, while TON values are rounded to the nearest 5 or 10. All data are the average of at least two experiments which were repeated if the values differed by more than 3%. Up to four other products were observed but could not be characterised. The MS data for the two most common minor products indicated that one was a monooxygenation product (M+16) while the mass of the second (M+14) suggested a carbonyl derivative.

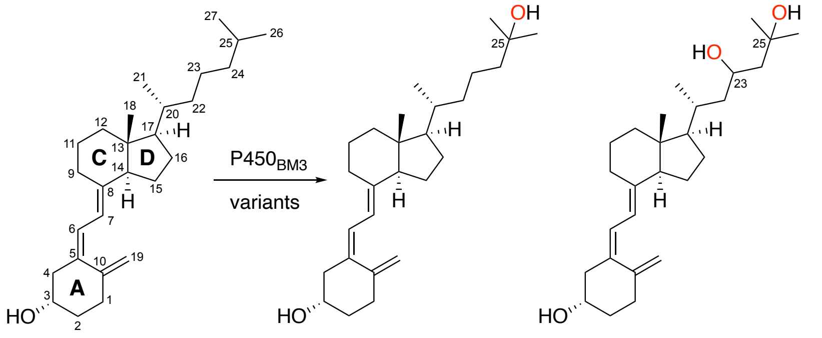

Variant	Mutations	1	2	Other	Conv.	TON
**M73**	R19/F87A/T268S	72%	9%	19%	39%	285
**M74**	R19/F87A/T268S/A328I	74%	3%	23%	36%	270
**M78**	K19/F87A/A82M/T260G/T268S	50%	3%	47%	18%	90
**M79**	R19/F87A/A82M/T260G/A328G	70%	3%	27%	37%	265
**M80**	R19/F87A/A82M/T260G/A184I	64%	3%	33%	32%	205
**M85**	R19/F87A/A82M/T260G/A184I/A328G	64%	8%	28%	30%	195
**M86**	R19/F87A/A82M/T260G/A184I/A328G/A330I	56%	9%	35%	30%	170
**M87**	R19/F87A/A82M/T260G/A184I/A328G/A330V	61%	8%	31%	25%	155
**M88**	R19/F87A/A82M/T260G/A184I/A328G/A330L	52%	10%	38%	29%	150
**M89**	R19/F87A/A82M/T260G/A184I/A328G/S72F	43%	6%	51%	19%	80
**M90**	R19/F87A/A82M/T260G/A184I/A328G/S72W	69%	11%	20%	54%	375
**M91**	F87A				<3%	
**M92**	F87A/A82M	72%		28%	28%	200
**M93**	F87A/A82M/A184I	30%		70%	26%	85
**M94**	F87A/A82M/T260G	38%	5%	57%	15%	60
**M95**	F87A/A82M/A184I/E435M	61%	21%	18%	53%	320
**M96**	F87A/A82M/A184I/T260G	75%	5%	20%	40%	300
**M97**	F87A/A82M/A184I/T260G/E435M	72%	5%	23%	50%	360
**M98**	F87A/A82M/A184I/T260G/L150P/M354G/E435M	19%	12%	69%	26%	50
**M99**	F87A/A82M/A184I/T260G/M354L/E435M	76%	4%	20%	71%	540
**M100**	F87A/A82M/A184I/T260G/S332A	57%	6%	37%	22%	130
**M101**	F87A/A82M/E435M	67%	20%	13%	66%	440
**M102**	F87A/A82M/S72A	65%	14%	21%	46%	300

Variant	Mutations	1	2	Other	Conv.	TON
**M103**	F87A/A82M/S72A/A184F/T260G/E435M	72%	5%	23%	42%	300
**M104**	F87A/A82M/S72A/A184I/T260G/E435T	70%	6%	24%	77%	540
**M105**	F87A/A82M/S72A/A184I	50%	32%	18%	52%	260
**M106**	F87A/A82M/S72A/A184I/E435M	71%	11%	18%	54%	390
**M107**	F87A/A82M/S72A/A184I/N239H/T260G/E435M	66%	28%	6%	81%	530
**M108**	F87A/A82M/S72A/A184I/T260A	38%	5%	57%	15%	55
**M109**	F87A/A82M/S72A/A184I/T260A/E435M	48%	9%	43%	20%	95
**M110**	F87A/A82M/S72A/A184I/T260G	63%	6%	31%	37%	235
**M111**	F87A/A82M/S72A/A184I/T260G/E435D	69%	7%	24%	35%	240
**M112**	F87A/A82M/S72A/A184I/T260G/E435H	66%	18%	16%	45%	295
**M113**	F87A/A82M/S72A/A184I/T260G/E435I	75%	14%	11%	83%	620
**M114**	F87A/A82M/S72A/A184I/T260G/E435I/V26H	30%	9%	61%	10%	30
**M115**	F87A/A82M/S72A/A184I/T260G/E435I/V26L	53%	5%	42%	14%	75
**M116**	F87A/A82M/S72A/A184I/T260G/E435I/V26M	67%	5%	28%	23%	155
**M117**	F87A/A82M/S72A/A184I/T260G/E435L	65%	5%	30%	29%	190
**M118**	F87A/A82M/S72A/A184I/T260G/E435M	73%	15%	12%	79%	570
**M119**	F87A/A82M/S72A/A184I/T260G/E435M/N319Y	75%	11%	14%	61%	460
**M120**	F87A/A82M/S72A/A184I/T260G/E435M/Q307H	15%	6%	79%	11%	15
**M121**	F87A/A82M/S72A/A184I/T260G/E435M/V26H	76%	5%	19%	47%	360
**M122**	F87A/A82M/S72A/A184I/T260G/E435M/V26M	76%	5%	19%	45%	345
**M123**	F87A/A82M/S72A/A184I/T260G/E435N	42%	9%	49%	17%	70
**M124**	F87A/A82M/S72A/A184I/T260G/E435Q	69%	12%	19%	49%	340
**M125**	F87A/A82M/S72A/A184I/T260G/E435R	49%	8%	43%	17%	85
**M126**	F87A/A82M/S72A/A184I/T260G/E435T/V26H	17%	6%	77%	9%	15
**M127**	F87A/A82M/S72A/A184I/T260G/E435T/V26L	9%	2%	89%	37%	35
**M128**	F87A/A82M/S72A/A184I/T260G/E435T/V26M	18%	10%	72%	10%	20
**M129**	F87A/A82M/S72A/A184I/T260G/E435W	57%	5%	38%	24%	140
**M130**	F87A/A82M/S72A/A184I/T260G/E435Y	57%	7%	36%	25%	140
**M131**	F87A/A82M/S72A/A184I/T260G/H171L/E435M	9%	4%	87%	10%	10
**M132**	F87A/A82M/S72A/A184I/T260G/H171L/L181F/E435M	15%	5%	80%	12%	20
**M133**	F87A/A82M/S72A/A184I/T260G/H171L/N239H/E435M	16%	6%	78%	12%	20
**M134**	F87A/A82M/S72A/A184I/T260G/H171L/Q403P	14%	6%	80%	9%	15
**M135**	F87A/A82M/S72A/A184I/T260G/L181F/N239H/E435M	43%	29%	28%	32%	140
**M136**	F87A/A82M/S72A/A184I/T260G/Q403P/E435M	23%	10%	67%	11%	25
**M137**	F87A/A82M/S72A/A184I/T260G/V26L/E435M	74%	5%	21%	46%	340
**M138**	F87A/A82M/S72A/A184I/T260G/V26L/H171L	15%	4%	81%	11%	15
**M139**	F87A/A82M/S72A/A184I/T260G/V26L/H171L/E435M	16%	6%	78%	12%	20
**M140**	F87A/A82M/S72A/A184I/T260G/V26L/N239H/E435M	77%	8%	15%	68%	530
**M141**	F87A/A82M/S72A/A184I/T260G/V26L/Q403P	9%	4%	87%	8%	5
**M142**	F87A/A82M/S72A/A184I/T260G/V26L/Y51F	69%	8%	23%	47%	325
**M143**	F87A/A82M/S72A/A184I/T260G/Y51F/H171L	14%	7%	79%	10%	15
**M144**	F87A/A82M/S72A/A184I/T260G/Y51F/Q403P	14%	8%	78%	10%	15
**M145**	F87A/A82M/S72A/A184L/T260G/E435I	69%	5%	26%	35%	245

Variant	Mutations	1	2	Other	Conv.	TON
**M146**	F87A/A82M/S72A/A184M/T260G/E435I	29%	9%	62%	12%	35
**M147**	F87A/A82M/S72A/A184M/T260G/E435M	64%	7%	29%	29%	185
**M148**	F87A/A82M/S72A/A184V/T260G/E435I	67%	5%	28%	34%	230
**M149**	F87A/A82M/S72A/E435M	45%	41%	14%	55%	245
**M150**	F87A/A82M/S72A/L181F/A184G/T260G	17%	6%	77%	11%	20
**M151**	F87A/A82M/S72A/T260G	47%	4%	49%	21%	100
**M152**	F87A/A82M/S72A/T260G/E435M	64%	10%	26%	42%	265
**M153**	F87A/A82M/T260G/E435M	73%	6%	21%	52%	380
**M154**	F87A/A82M/V178F/T260G/E435M	51%	5%	44%	31%	155
**M155**	F87A/A82M/V178W/T260G/E435M	51%	5%	44%	29%	145
**M156**	F87A/S72A/A184I/T260G/E435M	64%	7%	29%	24%	155
**M157**	F87A/S72A/A184I/T260G/E435T	34%	7%	59%	11%	35
**M158**	F87I/A82M	70%	7%	23%	32%	220
**M159**	F87I/A82M/A184I/E435M	61%	11%	28%	29%	175
**M160**	F87I/A82M/A184I/T260G	60%	6%	34%	25%	150
**M161**	F87I/A82M/A184I/T260G/E435I/S72V	85%		15%	54%	460
**M162**	F87I/A82M/A184I/T260G/E435I/S72W	62%	5%	33%	20%	125
**M163**	F87I/A82M/A184I/T260G/E435M	86%	4%	10%	71%	615
**M164**	F87I/A82M/A184I/T260G/E435M/S72V	81%	2%	17%	46%	375
**M165**	F87I/A82M/A184I/T260G/E435M/S72W	34%	5%	61%	11%	40
**M166**	F87I/A82M/E435M	71%	8%	21%	36%	255
**M167**	F87I/A82M/S72A	69%	9%	22%	34%	235
**M168**	F87I/A82M/S72A/A184I	34%	13%	53%	17%	60
**M169**	F87I/A82M/S72A/A184I/E435M	56%	14%	30%	25%	140
**M170**	F87I/A82M/S72A/A184I/N239H/T260G/E435I	82%	10%	8%	83%	680
**M171**	F87I/A82M/S72A/A184I/N239H/T260G/E435M	75%	13%	12%	79%	590
**M172**	F87I/A82M/S72A/A184I/T260G	80%	5%	15%	52%	420
**M173**	F87I/A82M/S72A/A184I/T260G/E435I	83%	8%	9%	83%	690
**M174**	F87I/A82M/S72A/A184I/T260G/E435I/L75S	84%		15%	71%	600
**M175**	F87I/A82M/S72A/A184I/T260G/E435I/M185T	79%		20%	61%	480
**M176**	F87I/A82M/S72A/A184I/T260G/E435I/M354F	13%	7%	80%	14%	20
**M177**	F87I/A82M/S72A/A184I/T260G/E435M	81%	10%	9%	83%	670
**M178**	F87I/A82M/S72A/A184I/T260G/E435M/L188S	76%	2%	22%	54%	410
**M179**	F87I/A82M/S72A/A184I/T260G/E435M/L29A	82%		17%	68%	555
**M180**	F87I/A82M/S72A/A184I/T260G/E435M/L29M	86%	4%	10%	80%	685
**M181**	F87I/A82M/S72A/A184I/T260G/E435M/L75S	14%	6%	80%	14%	20
**M182**	F87I/A82M/S72A/A184I/T260G/E435M/L75T	13%	6%	81%	15%	20
**M183**	F87I/A82M/S72A/A184I/T260G/E435M/M354F	85%	3%	12%	79%	670
**M184**	F87I/A82M/S72A/E435M	71%	16%	13%	48%	340
**M185**	F87I/A82M/S72A/T260G	67%	4%	29%	28%	185
**M186**	F87I/A82M/S72A/T260G/E435M	76%	18%	6%	75%	565
**M187**	F87I/A82M/S72A/V178F/T260G/E435I	70%	2%	28%	43%	295
**M188**	F87I/A82M/S72A/V178F/T260G/E435M	75%	6%	19%	55%	410

Variant	Mutations	1	2	Other	Conv.	TON
**M189**	F87I/A82M/S72A/V178W/T260G/E435I	80%	2%	18%	59%	475
**M190**	F87I/A82M/S72A/V178W/T260G/E435M	80%	8%	12%	75%	605
**M191**	F87I/A82M/T260G	36%	5%	59%	15%	55
**M192**	F87I/A82M/T260G/E435M	80%	6%	14%	50%	400
**M193**	F87S/A82M/S72A/A184I/T260G/E435M	76%	7%	17%	70%	525
**M194**	F87T/A82M/S72A/A184I/T260G/E435I	78%	9%	13%	70%	550
**M195**	F87T/A82M/S72A/A184I/T260G/E435M	74%	14%	12%	76%	560
**M196**	F87V/A82M/S72A/A184I/N239H/T260G/E435I	17%	9%	74%	10%	15
**M197**	F87V/A82M/S72A/A184I/N239H/T260G/E435M	50%	41%	9%	71%	360
**M198**	F87V/A82M/S72A/A184I/T260G/E435I	50%	42%	8%	83%	410
**M199**	F87V/A82M/S72A/A184I/T260G/E435M	49%	40%	11%	77%	380
**M200**	K69I/F87A/A82M/A184I/T260G/E435M	15%	8%	77%	17%	25
**M201**	K69R/F87A/A82M/A184I/T260G/E435M	20%	11%	69%	26%	50
**M202**	F87I/A82M/S72A/A184I/M185T/T260G/E435M/L29M	82%	4%	14%	53%	430
**M203**	L29M/S72A/L75S/A82M/F87T/A184I/T260G/E435I	22%	8%	70%	14%	30
**M204**	L29M/S72A/V78M/A82M/F87T/A184I/T260G/E435I	48%	6%	46%	20%	95
**M205**	R19/F87A/A82M/A184I/T260G/E435I	16%	6%	78%	8%	15
**M206**	R19/F87A/A82M/A184I/T260G/E435M	61%	5%	34%	24%	145
**M207**	F87T/A82M/S72A/A184I/T260G/E435M/M185T	69%	7%	24%	36%	250
**M208**	F87T/A82M/S72A/A184I/T260G/E435I/L75S	44%	6%	50%	19%	85
**M209**	F87T/A82M/S72A/A184I/T260G/E435I/V78M	78%	8%	14%	62%	480
**M210**	F87A/A82M/S72A/A184I/T260G/E435M/Y51F	76%	13%	11%	67%	510
**M211**	R19/F87I/A82M/A184I/T260G	34%	5%	61%	14%	50
**M212**	R19/F87T/A82M/A184I/T260G	68%	12%	20%	62%	420
**M213**	R19/F87V/A82M/A184I/T260G	66%	12%	22%	56%	365
**M214**	R19/F87A/A82M/A184I/T260G/E435T	64%	6%	30%	42%	270
**M215**	R19/F87A/A82M/A184I/T260G/S72A	66%	23%	11%	69%	455

Table S5. Activity and product selectivity for the oxidation of vitamin D_2 (VD_2) to 25-hydroxyVD_2 (**3**) and 24,25-dihydroxyVD_2 (**4**) catalysed by $P450_{BM3}$ variants (K19 = H171L/Q307H/N319Y. R19 = R47L/Y51F/H171L/Q307H/N319Y). The substrate-to-enzyme concentration ratio was 1000:1 (2 mM VD_2, 2 μM $P450_{BM3}$ enzyme). Conv. is the percentage of substrate converted to products. TON is the turnover number of the variant for the formation of **3**. All percentages are rounded to the nearest integer, while TON values are rounded to the nearest 5 or 10. All data are the average of at least two experiments which were repeated if the values differed by more than 3%. Up to three other products were observed but could not be characterised. The MS data (M+16) for the most common minor product indicated that it was a monooxygenation product.

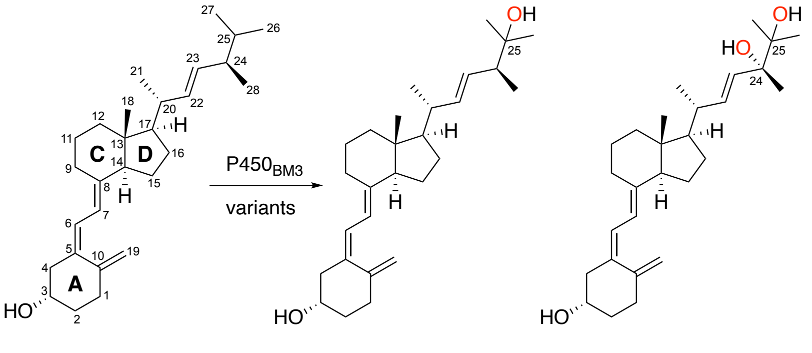

Ergocalciferol (vitamin D_2)
25-Hydroxyergocalciferol, **3** [25(OH)VD_2, ercalcidiol]
24,25-Dihydroxyergocalciferol, **4** [24,25-$(OH)_2VD_2$]

Variant	Mutations	3	4	Other	Conv.	TON
**M92**	F87A/A82M	57%		43%	19%	105
**M102**	F87A/A82M/S72A	79%	5%	16%	25%	200
**M101**	F87A/A82M/E435M	92%	4%	4%	57%	530
**M149**	F87A/A82M/S72A/E435M	72%	17%	11%	55%	400
**M96**	F87A/A82M/A184I/T260G	62%		38%	17%	110
**M110**	F87A/A82M/A184I/T260G/S72A	59%	6%	35%	31%	185
**M97**	F87A/A82M/A184I/T260G/E435M	94%		6%	35%	330
**M118**	F87A/A82M/S72A/A184I/T260G/E435M	96%	2%	2%	71%	685
**M113**	F87A/A82M/S72A/A184I/T260G/E435I	96%	2%	2%	77%	750
**M199**	F87V/A82M/S72A/A184I/T260G/E435M	53%	23%	24%	75%	395
**M198**	F87V/A82M/S72A/A184I/T260G/E435I	50%	46%	4%	80%	400
**M177**	F87I/A82M/S72A/A184I/T260G/E435M	88%	12%		81%	720
**M173**	F87I/A82M/S72A/A184I/T260G/E435I	92%	4%	4%	88%	810
**M73**	R19/F87A/T268S	62%		38%	21%	130
**M74**	R19/F87A/T268S/A328I	66%	2%	32%	27%	180
**M78**	K19/F87A/A82M/T260G/T268S	53%	2%	45%	12%	60
**M79**	R19/F87A/A82M/T260G/A328G	79%		21%	33%	255
**M80**	R19/F87A/A82M/T260G/A184I	60%	4%	36%	17%	105
**M85**	R19/F87A/A82M/T260G/A184I/A328G	67%	2%	31%	21%	145
**M86**	R19/F87A/A82M/T260G/A184I/A328G/A330I	59%	3%	38%	16%	95
**M87**	R19/F87A/A82M/T260G/A184I/A328G/A330V	50%	3%	47%	14%	70
**M88**	R19/F87A/A82M/T260G/A184I/A328G/A330L	73%		27%	18%	135
**M89**	R19/F87A/A82M/T260G/A184I/A328G/S72F	51%		49%	17%	85

Variant	Mutations	3	4	Other	Conv.	TON
**M90**	R19/F87A/A82M/T260G/A184I/A328G/S72W	84%	3%	13%	49%	410
**M91**	F87A				<3%	
**M93**	F87A/A82M/A184I	55%		45%	10%	55
**M94**	F87A/A82M/T260G	59%		41%	4%	25
**M95**	F87A/A82M/A184I/E435M	82%		18%	37%	305
**M98**	F87A/A82M/A184I/T260G/L150P/M354G/E435M	3%		97%	6%	
**M99**	F87A/A82M/A184I/T260G/M354L/E435M	86%		14%	36%	315
**M100**	F87A/A82M/A184I/T260G/S332A	60%		39%	15%	90
**M103**	F87A/A82M/S72A/A184F/T260G/E435M	89%		10%	30%	270
**M104**	F87A/A82M/S72A/A184G/T260G/E435T	94%		5%	59%	550
**M105**	F87A/A82M/S72A/A184I	70%	10%	20%	23%	160
**M106**	F87A/A82M/S72A/A184I/E435M	93%		7%	38%	350
**M107**	F87A/A82M/S72A/A184I/T260G/N239H/E435M	94%	3%	3%	73%	690
**M108**	F87A/A82M/S72A/A184I/T260A	50%		49%	8%	40
**M109**	F87A/A82M/S72A/A184I/T260A/E435M	82%		17%	21%	175
**M111**	F87A/A82M/S72A/A184I/T260G/E435D	88%		11%	34%	300
**M112**	F87A/A82M/S72A/A184I/T260G/E435H	86%	3%	11%	31%	265
**M114**	F87A/A82M/S72A/A184I/T260G/E435I/V26H	25%	4%	71%	3%	10
**M115**	F87A/A82M/S72A/A184I/T260G/E435I/V26L	38%	6%	56%	5%	20
**M116**	F87A/A82M/S72A/A184I/T260G/E435I/V26M	77%	2%	21%	13%	100
**M117**	F87A/A82M/S72A/A184I/T260G/E435L	82%	2%	16%	15%	125
**M119**	F87A/A82M/S72A/A184I/T260G/E435M/N319Y	93%	2%	5%	56%	520
**M120**	F87A/A82M/S72A/A184I/T260G/E435M/Q307H	8%	4%	88%	3%	2
**M121**	F87A/A82M/S72A/A184I/T260G/E435M/V26H	88%		11%	32%	285
**M122**	F87A/A82M/S72A/A184I/T260G/E435M/V26M	90%		9%	34%	305
**M123**	F87A/A82M/S72A/A184I/T260G/E435N	50%		49%	8%	40
**M124**	F87A/A82M/S72A/A184I/T260G/E435Q	87%		12%	33%	290
**M125**	F87A/A82M/S72A/A184I/T260G/E435R	51%	3%	46%	7%	35
**M126**	F87A/A82M/S72A/A184I/T260G/E435T/V26H	7%		92%	4%	2
**M127**	F87A/A82M/S72A/A184I/T260G/E435T/V26L	29%	7%	64%	5%	15
**M128**	F87A/A82M/S72A/A184I/T260G/E435T/V26M	8%	6%	86%	4%	5
**M129**	F87A/A82M/S72A/A184I/T260G/E435W	81%		18%	20%	160
**M130**	F87A/A82M/S72A/A184I/T260G/E435Y	76%		23%	14%	105
**M131**	F87A/A82M/S72A/A184I/T260G/H171L/E435M	17%		83%	5%	10
**M132**	F87A/A82M/S72A/A184I/T260G/H171L/L181F/E435M	12%	4%	84%	5%	5
**M133**	F87A/A82M/S72A/A184I/T260G/H171L/N239H/E435M	6%	5%	89%	5%	5
**M134**	F87A/A82M/S72A/A184I/T260G/H171L/Q403P	8%		91%	4%	5
**M135**	F87A/A82M/S72A/A184I/T260G/L181F/N239H/E435M	64%	14%	22%	16%	100
**M136**	F87A/A82M/S72A/A184I/T260G/Q403P/E435M	29%		71%	3%	9
**M137**	F87A/A82M/S72A/A184I/T260G/V26L/E435M	86%		13%	20%	170
**M138**	F87A/A82M/S72A/A184I/T260G/V26L/H171L	13%		87%	5%	5

Variant	Mutations	3	4	Other	Conv.	TON
**M139**	F87A/A82M/S72A/A184I/T260G/V26L/H171L/E435M	23%		77%	6%	15
**M140**	F87A/A82M/S72A/A184I/T260G/V26L/N239H/E435M	91%		9%	73%	665
**M141**	F87A/A82M/S72A/A184I/T260G/V26L/Q403P	12%	2%	86%	3%	5
**M142**	F87A/A82M/S72A/A184I/T260G/V26L/Y51F	89%	2%	9%	36%	325
**M143**	F87A/A82M/S72A/A184I/T260G/Y51F/H171L	11%		89%	4%	5
**M144**	F87A/A82M/S72A/A184I/T260G/Y51F/Q403P	11%		89%	4%	5
**M145**	F87A/A82M/S72A/A184L/T260G/E435I	87%		13%	24%	215
**M146**	F87A/A82M/S72A/A184M/T260G/E435I	32%	4%	64%	5%	15
**M147**	F87A/A82M/S72A/A184M/T260G/E435M	86%	2%	12%	22%	190
**M148**	F87A/A82M/S72A/A184V/T260G/E435I	88%		11%	26%	230
**M150**	F87A/A82M/S72A/L181F/A184G/T260G	11%	7%	82%	4%	5
**M151**	F87A/A82M/S72A/T260G	45%		55%	16%	70
**M152**	F87A/A82M/S72A/T260G/E435M	93%	2%	5%	47%	435
**M153**	F87A/A82M/T260G/E435M	95%	2%	3%	28%	265
**M154**	F87A/A82M/V178F/T260G/E435M	54%		45%	12%	65
**M155**	F87A/A82M/V178W/T260G/E435M	70%		29%	18%	120
**M156**	F87A/S72A/A184I/T260G/E435M	76%	3%	21%	16%	120
**M157**	F87A/S72A/A184I/T260G/E435T	25%	8%	67%	6%	15
**M158**	F87I/A82M	76%	2%	22%	23%	175
**M159**	F87I/A82M/A184I/E435M	73%	5%	22%	24%	170
**M160**	F87I/A82M/A184I/T260G	67%	2%	31%	14%	95
**M161**	F87I/A82M/A184I/T260G/E435I/S72V	91%		9%	41%	375
**M162**	F87I/A82M/A184I/T260G/E435I/S72W	69%	2%	29%	12%	85
**M163**	F87I/A82M/A184I/T260G/E435M	90%	2%	8%	59%	530
**M164**	F87I/A82M/A184I/T260G/E435M/S72V	89%		10%	38%	340
**M165**	F87I/A82M/A184I/T260G/E435M/S72W	30%		70%	5%	15
**M166**	F87I/A82M/E435M	74%	5%	21%	25%	185
**M167**	F87I/A82M/S72A	78%		22%	25%	195
**M168**	F87I/A82M/S72A/A184I	44%	2%	54%	10%	45
**M169**	F87I/A82M/S72A/A184I/E435M	65%	9%	26%	22%	140
**M170**	F87I/A82M/S72A/A184I/N239H/T260G/E435I	88%	10%	2%	89%	780
**M171**	F87I/A82M/S72A/A184I/N239H/T260G/E435M	79%	14%	7%	78%	615
**M172**	F87I/A82M/S72A/A184I/T260G	85%		14%	37%	315
**M174**	F87I/A82M/S72A/A184I/T260G/E435I/L75S	89%	3%	8%	58%	515
**M175**	F87I/A82M/S72A/A184I/T260G/E435I/M185T	91%		9%	55%	500
**M176**	F87I/A82M/S72A/A184I/T260G/E435I/M354F	5%		95%	5%	2
**M178**	F87I/A82M/S72A/A184I/T260G/E435M/L188S	86%	2%	12%	44%	375
**M179**	F87I/A82M/S72A/A184I/T260G/E435M/L29A	90%		10%	37%	340
**M180**	F87I/A82M/S72A/A184I/T260G/E435M/L29M	94%	2%	4%	78%	740
**M181**	F87I/A82M/S72A/A184I/T260G/E435M/L75S	5%		95%	8%	5
**M182**	F87I/A82M/S72A/A184I/T260G/E435M/L75T			100%	8%	

Variant	Mutations	3	4	Other	Conv.	TON
**M183**	F87I/A82M/S72A/A184I/T260G/E435M/M354F	91%	2%	7%	77%	700
**M184**	F87I/A82M/S72A/E435M	59%	18%	23%	39%	230
**M185**	F87I/A82M/S72A/T260G	81%		18%	25%	205
**M186**	F87I/A82M/S72A/T260G/E435M	89%	3%	8%	69%	610
**M187**	F87I/A82M/S72A/V178F/T260G/E435I	87%		13%	39%	340
**M188**	F87I/A82M/S72A/V178F/T260G/E435M	90%		10%	51%	465
**M189**	F87I/A82M/S72A/V178W/T260G/E435I	92%		8%	58%	535
**M190**	F87I/A82M/S72A/V178W/T260G/E435M	86%	5%	9%	72%	620
**M191**	F87I/A82M/T260G	44%	3%	53%	7%	35
**M192**	F87I/A82M/T260G/E435M	82%	2%	16%	22%	180
**M193**	F87S/A82M/S72A/A184I/T260G/E435M	89%		11%	48%	430
**M194**	F87T/A82M/S72A/A184I/T260G/E435I	92%	2%	6%	75%	695
**M195**	F87T/A82M/S72A/A184I/T260G/E435M	91%		9%	70%	630
**M196**	F87V/A82M/S72A/A184I/N239H/T260G/E435I	4%		96%	3%	
**M197**	F87V/A82M/S72A/A184I/N239H/T260G/E435M	44%	28%	28%	68%	295
**M200**	K69I/F87A/A82M/A184I/T260G/E435M	4%	2%	94%	7%	5
**M201**	K69R/F87A/A82M/A184I/T260G/E435M	14%	2%	84%	6%	10
**M202**	F87I/A82M/S72A/A184I/M185T/T260G/E435M/L29M	88%		12%	48%	420
**M203**	L29M/S72A/L75S/A82M/F87T/A184I/T260G/E435I	10%	6%	84%	8%	10
**M204**	L29M/S72A/V78M/A82M/F87T/A184I/T260G/E435I	28%	3%	69%	6%	20
**M205**	R19/F87A/A82M/A184I/T260G/E435I	2%		98%	3%	
**M206**	R19/F87A/A82M/A184I/T260G/E435M	75%	6%	19%	17%	130
**M207**	F87T/A82M/S72A/A184I/T260G/E435M/M185T	86%		14%	33%	285
**M208**	F87T/A82M/S72A/A184I/T260G/E435I/L75S	44%	5%	51%	10%	45
**M209**	F87T/A82M/S72A/A184I/T260G/E435/V78M	84%	3%	13%	29%	245
**M210**	F87A/A82M/S72A/A184I/T260G/E435M/Y51F	93%	2%	5%	57%	525
**M211**	R19/F87I/A82M/A184I/T260G	38%	4%	58%	8%	30
**M212**	R19/F87T/A82M/A184I/T260G	73%	15%	12%	47%	340
**M213**	R19/F87V/A82M/A184I/T260G	75%	13%	12%	39%	295
**M214**	R19/F87A/A82M/A184I/T260G/E435T	72%	16%	12%	40%	285
**M215**	R19/F87A/A82M/A184I/T260G/S72A	87%	6%	7%	58%	505

Table S6. Activity and selectivity for the oxidation of VD_3 with the F87I/A82M/A184I/T260G/S72A/E435I variant (M173) of $P450_{BM3}$. The screening scale reaction mixture (0.5 mL) contained 2 µM enzyme, 4 U/mL GDH, and 40 µM $NADP^+$. Conv. is the percentage of substrate converted to products. TON is the turnover number for the formation of 25(OH)VD_3 (**1**).

Entry	$[VD_3]$/mM	$[VD_3]$:$[P450_{BM3}]$	**1**	**Conv.**	**TON**
**1**	2	1000:1	83%	83%	690
**2**	5	2500:1	62%	92%	1440
**3**	6	3000:1	87%	86%	2230
**4**	7	3500:1	81%	78%	2200
**5**	8	4000:1	81%	76%	2440
**6**	9	4500:1	83%	74%	2780
**7**	10	5000:1	77%	72%	2750

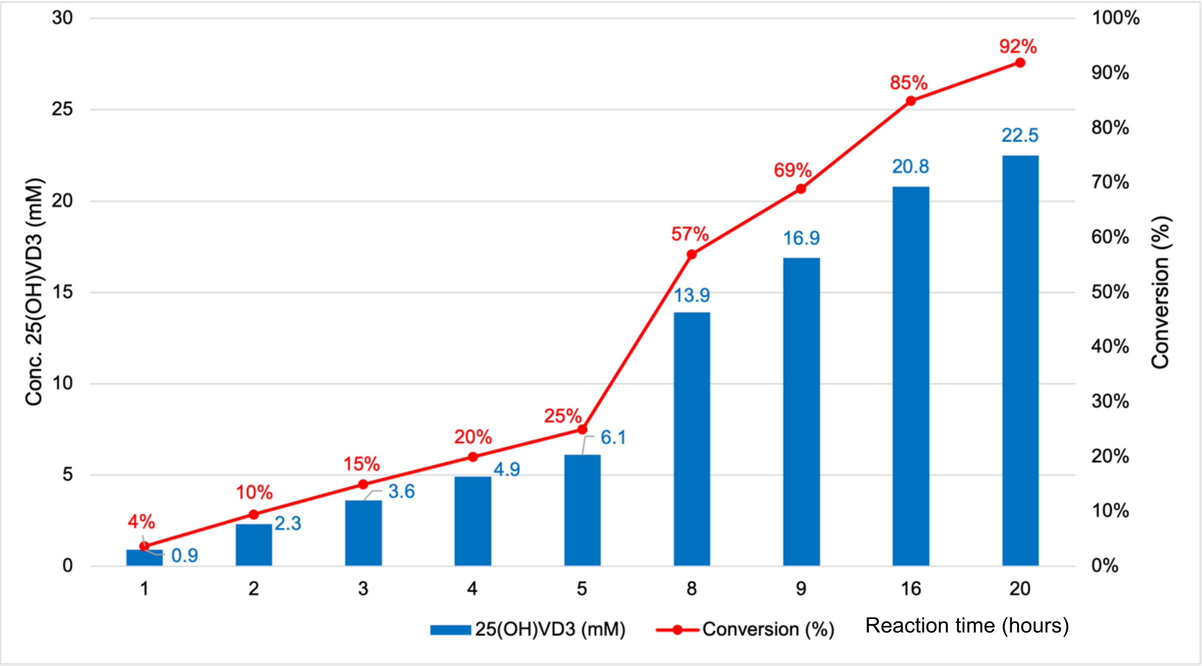

Figure S2. Time course for the conversion of vitamin D_3 and formation of 25-hydroxy-vitamin D_3 (**1**) in a 1-L scale reaction containing 10 g (26 mmol) of vitamin D_3 and 5 µmol of the F87I/A82M/A184I/T260G/S72A/E435I variant (M173) of $P450_{BM3}$. After stirring (400 rpm) with aeration (1.5 L/min) at ambient temperature for 20 hours, the reaction reached 92% conversion, from which 6.62 g of 25-hydroxy-vitamin D_3 (**1**) was isolated via silica gel column chromatography (69.1% yield based on VD_3 converted).

S4. Product characterisation

1. 25-Hydroxy-vitamin D_3

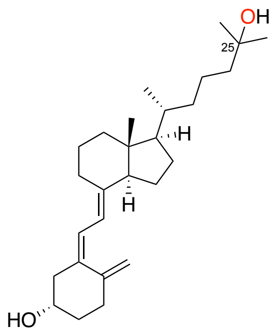

A preparative scale reaction was conducted to isolate and characterise product **1** using the variant S72A/F87A/A82M/A184I/T260G/E435I. The 100 mL reaction mixture in 200 mM potassium phosphate buffer, pH 7.9, contained 2 µM of this enzyme variant, 384 mg VD_3 (10 mM, 200 mM stock solution in methanol), 4 U/mL glucose dehydrogenase (GDH, 4 U/µL stock solution), 100 mM glucose (1 M stock solution) and 10 mM methyl-β-cyclodextrin (200 mM stock solution). $NADP^+$ monosodium salt (40 µM; 4 mM stock solution) was added to initiate the reaction. After stirring for 24 h the reaction reached 64% conversion with 87% selectivity for **1**. The reaction mixture was extracted three times with ethyl acetate. The organic extracts were combined and washed with water and then brine, dried with Na_2SO_4, and solvent was removed by rotary evaporation. The crude mixture was purified by silica gel column chromatography, eluting with mixtures of petroleum ether (bp 40–60 °C) and ethyl acetate (5:1 to 3:1 to 1:1) to give product **1** as a white solid (112 mg, 44% yield based on the amount of VD_3 converted). The assignment of **1** was consistent with literature data.[45]

^{1}H NMR (700 MHz, $CDCl_3$) δ 6.22 (d, J = 11.0 Hz, 1H), 6.03 (d, J = 11.5 Hz, 1H), 5.04 (d, J = 2.5 Hz, 1H), 4.81 (d, J = 2.5 Hz, 1H), 3.97 – 3.90 (m, 1H), 2.84 – 2.79 (m, 1H), 2.56 (dd, J = 13.0, 4.0 Hz, 1H), 2.39 (m, 1H), 2.28 (dd, J = 13.0, 7.5 Hz, 1H), 2.19 – 2.14 (m, 1H), 2.02 – 1.96 (m, 2H), 1.94 – 1.89 (m, 1H), 1.86 (m, 1H), 1.67 (m, 3H), 1.54 – 1.51 (m, 1H), 1.49 – 1.44 (m, 3H), 1.40 – 1.36 (m, 3H), 1.32 – 1.26 (m, 3H), 1.21 (s, 8H), 1.08 – 1.03 (m, 1H), 0.93 (d, J = 6.5 Hz, 3H), 0.54 (s, 3H). **^{13}C NMR** (176 MHz, $CDCl_3$) δ 145.2, 142.4, 135.2, 122.6, 117.7, 112.5, 71.3, 69.3, 56.7, 56.5, 46.1, 46.0, 44.5, 40.7, 36.5, 36.2, 35.3, 32.1, 29.5, 29.4, 29.1, 27.8, 23.7, 22.4, 21.0, 18.9, 12.1. **HRMS** (ESI^+): Calc'd for $C_{27}H_{45}O_2^+$ $[M+H]^+$: 401.3414, found: 401.3414.

2. 23,25-Dihydroxy-vitamin D_3

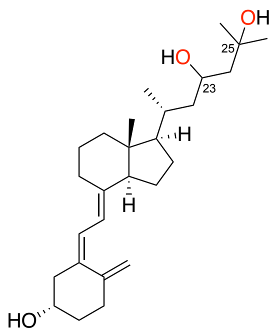

A preparative scale reaction was conducted to isolate and characterise product **2** using the variant S72A/F87A/A82M/A184I/T260G/E435I. The 100 mL reaction mixture in 200 mM potassium phosphate buffer, pH 7.9, contained 2 µM of this enzyme variant, 384 mg VD_3 (10 mM, 200 mM solution stock in methanol), 4 U/mL glucose dehydrogenase (GDH, 4 U/µL stock), 100 mM glucose (1 M stock solution) and 10 mM methyl-β-cyclodextrin (200 mM stock solution). $NADP^+$ monosodium salt (40 µM, 4 mM stock solution) was added to initiate the reaction. After stirring for 24 h the reaction reached 64% conversion with 8% selectivity for **2**. The reaction mixture was extracted three times with ethyl acetate. The organic extracts were combined and washed with water and then brine, dried with Na_2SO_4, and solvent was removed by rotary evaporation. The crude mixture was purified by silica gel column chromatography, eluting with mixtures of petroleum ether (bp 40–60 °C) and ethyl acetate (5:1 to 3:1 to 1:1) to give product **2** as a white solid (23 mg, 9% yield based on the amount of VD_3 converted). The assignment of **2** was consistent with literature data.[46]

^{1}H NMR (700 MHz, $CDCl_3$) δ 6.23 (d, *J* = 11.0 Hz, 1H), 6.03 (d, *J* = 11.0,z Hz, 1H), 5.06 – 5.03 (d, *J* = 2.5 Hz, 1H), 4.81 (d, *J* = 2.5 Hz, 1H), 4.10 (m, 1H), 3.95 (m, 1H), 2.82 (dd, *J* = 12.0, 4.0 Hz, 1H), 2.60 – 2.55 (m, 1H), 2.40 (m, 1H), 2.29 (dd, *J* = 13.5, 8.0 Hz, 1H), 2.18 (m, 1H), 2.02 – 1.97 (m, 2H), 1.95 – 1.88 (m, 2H), 1.71 – 1.65 (m, 3H), 1.57 – 1.49 (m, 6H), 1.32 – 1.27 (m, 11H), 0.97 (d, *J* = 6.0 Hz, 3H), 0.56 (s, 3H). **^{13}C NMR** (101 MHz, $CDCl_3$) δ 145.3, 142.1, 135.4, 122.5, 117.8, 112.5, 72.0, 69.3, 68.5, 57.4, 56.4, 47.6, 46.1, 46.0, 45.1, 40.7, 35.3, 34.1, 32.4, 32.1, 29.1, 28.1, 27.9, 23.7, 22.4, 19.5, 12.2. **HRMS** (ESI+): Calc'd for $C_{27}H_{44}NaO_3^+$ $[M+Na]^+$: 439.3183, found: 439.3183.

3. 25-Hydroxy-vitamin D_2

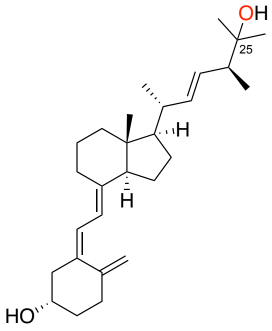

A preparative scale reaction was conducted to isolate and characterise product **3** using variant S72A/F87I/A82M/A184I/T260G/E435I which showed 88% conversion and 92% selectivity for **3** in 24 h. The 100 mL reaction mixture in 200 mM potassium phosphate buffer, pH 7.9, contained 2 μM of this enzyme variant, 159 mg VD_2 (4 mM, 200 mM solution stock in ethanol), 4 U/mL glucose dehydrogenase (GDH, 4 U/μL stock), 100 mM glucose (1 M stock) and 10 mM methyl-β-cyclodextrin (200 mM stock solution). $NADP^+$ monosodium salt (40 μM, 4 mM stock solution) was added to initiate the reaction. After stirring for 24 h the reaction mixture was extracted three times with ethyl acetate. The organic extracts were combined and washed with water and then brine, dried with Na_2SO_4, and solvent was removed by rotary evaporation. The crude mixture was purified by silica gel column chromatography, eluting with mixtures of petroleum ether (bp 40–60 °C) and ethyl acetate (5:1 to 3:1 to 1:1) to give product **3** as a white solid (95 mg, 66% yield based on the amount of VD_2 converted). The assignment of **3** was consistent with literature data.[47]

^{1}H NMR (600 MHz, CD_3OD) δ 6.22 (d, J = 11.0 Hz, 1H), 6.03 (d, J = 11.0 Hz, 1H), 5.35 (dd, J = 15.5, 8.0 Hz, 1H), 5.27 (dd, J = 15.0, 8.5 Hz, 1H), 5.03 (s, 1H), 4.77 – 4.73 (s, 1H), 3.76 (tt, J = 9.0, 4.0 Hz, 1H), 2.86 (dd, J = 12.0, 4.0 Hz, 1H), 2.57 – 2.51 (m, 1H), 2.40 (dt, J = 13.5, 5.0 Hz, 1H), 2.23 – 2.16 (m, 1H), 2.15 – 1.98 (m, 6H), 1.77 – 1.66 (m, 3H), 1.59 – 1.51 (m, 2H), 1.50 – 1.42 (m, 2H), 1.39 – 1.31 (m, 3H), 1.14 (s, 3H), 1.10 (s, 3H), 1.04 (d, J = 6.5 Hz, 3H), 1.00 (d, J = 7.0 Hz, 3H), 0.57 (s, 3H). **^{13}C NMR** (151 MHz, MeOD) δ 145.6, 141.0, 137.0, 136.0, 130.0, 121.3, 117.7, 111.4, 71.9, 69.2, 56.4, 56.2, 47.8, 45.7, 45.4, 40.5, 40.4, 35.2, 32.3, 28.6, 27.6, 27.0, 24.7, 23.2, 21.9, 20.1, 14.4, 11.4. **HRMS** (ESI+): Calc'd for $C_{28}H_{45}O_2^+$ $[M+H]^+$: 413.3414, found: 413.3399.

4. 24,25-Dihydroxy-vitamin D_2

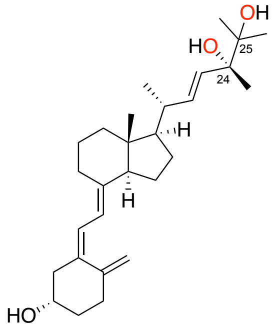

A preparative scale reaction was conducted to isolate and characterise product **4** using variant S72A/F87I/A82M/A184I/T260G/E435I which showed 88% conversion and 4% selectivity for **4** after 24 h. The 100 mL reaction mixture in 200 mM potassium phosphate buffer, pH 7.9, contained 2 µM of this variant, 159 mg vitamin D_2 (4 mM, 200 mM solution stock in ethanol), 4 U/mL glucose dehydrogenase (GDH, 4 U/µL stock solution), 100 mM glucose (1 M stock solution) and 10 mM methyl-β-cyclodextrin (200 mM stock solution). $NADP^+$ monosodium salt (40 µM, 4 mM stock solution) was added to initiate the reaction. After stirring for 24 h the reaction mixture was extracted three times with ethyl acetate. The organic extracts were combined and washed with water and then brine, dried with Na_2SO_4, and then solvent was removed by rotary evaporation. The crude mixture was purified by silica gel column chromatography, eluting with mixtures of petroleum ether (bp 40–60 °C) and ethyl acetate (5:1 to 3:1 to 1:1) to give **4** as a white solid (10 mg, 7% based on the amount of VD_2 converted). The assignment of **4** was consistent with literature data.[47,48]

^{1}H NMR (400 MHz, CD_3OD) δ 6.23 (d, J = 11.0 Hz, 1H), 6.04 (d, J = 11.0 Hz, 1H), 5.62 (d, J = 15.5 Hz, 1H), 5.57 – 5.50 (m, 1H), 5.07 – 5.03 (m, 1H), 4.76 (dd, J = 3.0, 1.0 Hz, 1H), 3.77 (tt, J = 9.0, 4.0 Hz, 1H), 2.88 (dd, J = 12.0, 4.0 Hz, 1H), 2.58 – 2.51 (m, 1H), 2.42 (dt, J = 13.5, 5.0 Hz, 1H), 2.18 – 2.05 (m, 6H), 1.76 (dt, J = 9.0, 2.5 Hz, 3H), 1.68 – 1.31 (m, 7H), 1.25 (d, J = 1.0 Hz, 3H), 1.19 (s, 3H), 1.17 (s, 3H), 1.07 (d, J = 6.5 Hz, 3H), 0.60 (s, 3H). **^{13}C NMR** (101 MHz, MeOD) δ 147.0, 142.4, 137.4, 136.5, 133.0, 122.6, 119.0, 112.7, 77.9, 75.8, 70.6, 57.7, 57.6, 47.0, 46.9, 41.8, 36.6, 33.6, 29.9, 29.1, 25.3, 25.2, 24.5, 23.2, 23.0, 21.3, 14.5, 12.6. **HRMS** (ESI+) Calc'd for $C_{28}H_{44}O_3Na^+$ [$M+Na^+$]: 451.3183, found: 451.3180.

1 – ^{1}H NMR (500 MHz, $CDCl_3$)

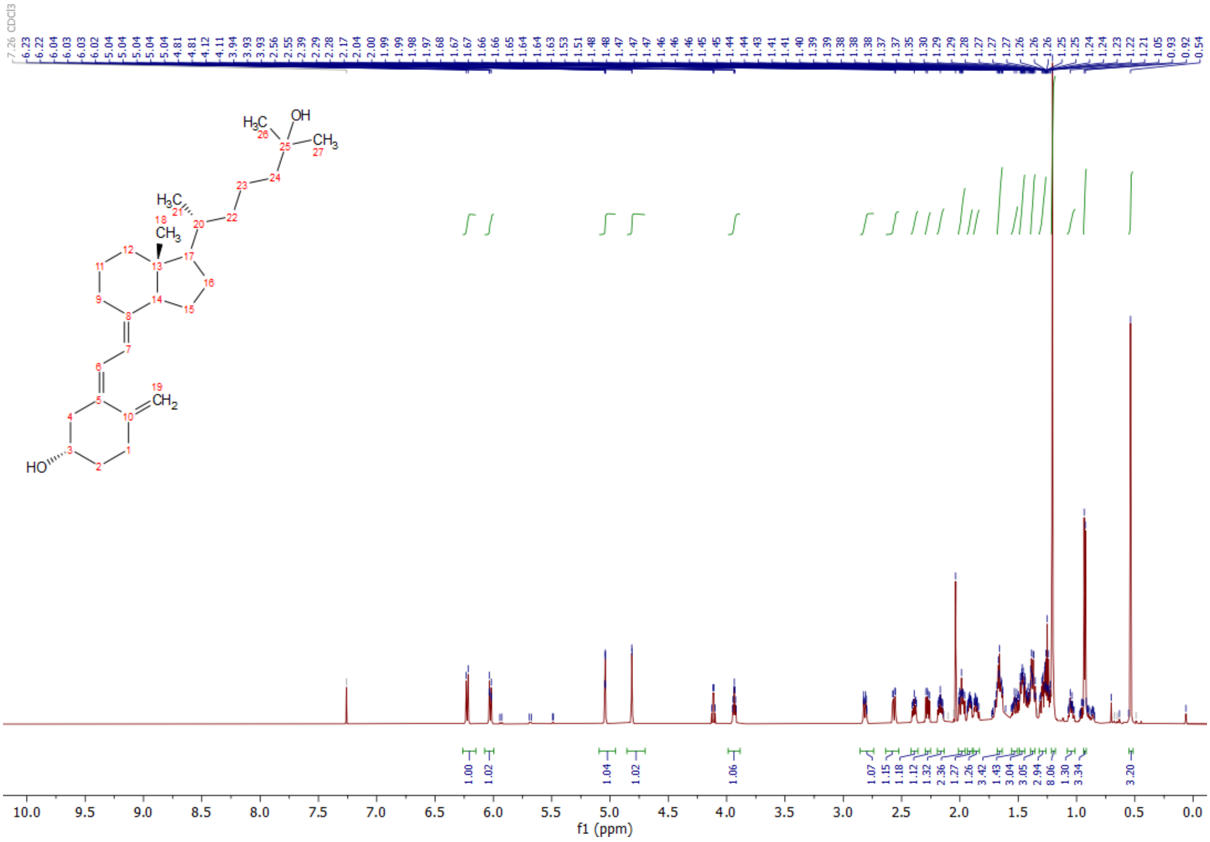

1 – ^{13}C NMR (126 MHz, $CDCl_3$)

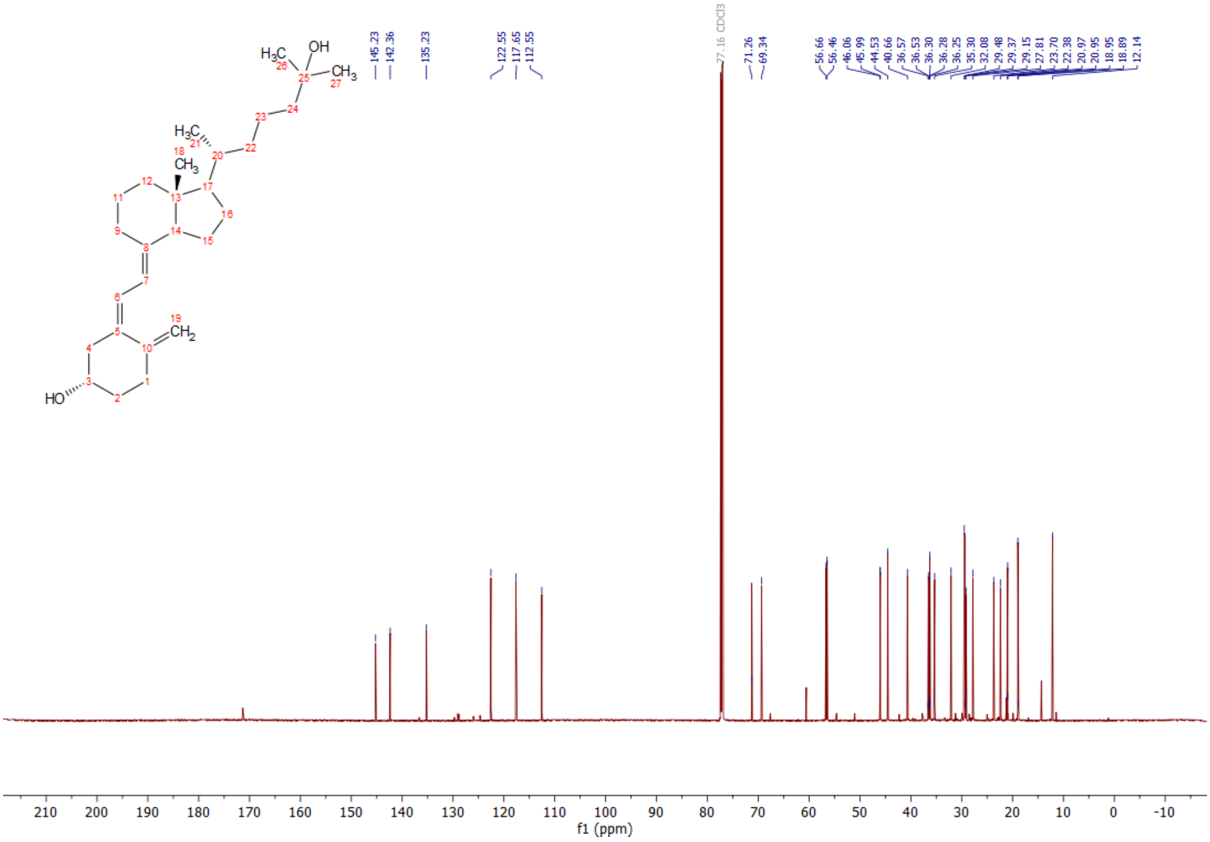

2 – ^{1}H NMR (500 MHz, $CDCl_3$)

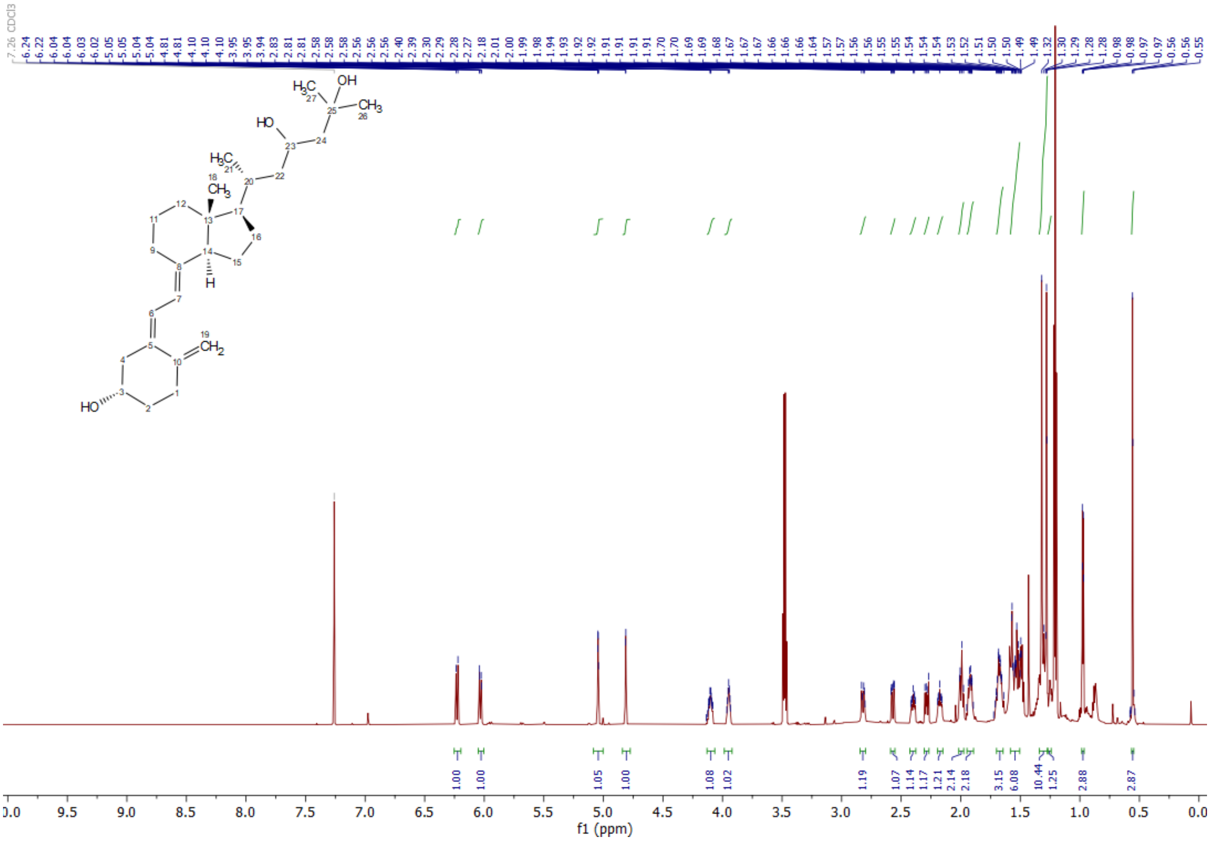

2 – ^{13}C NMR (101 MHz, $CDCl_3$)

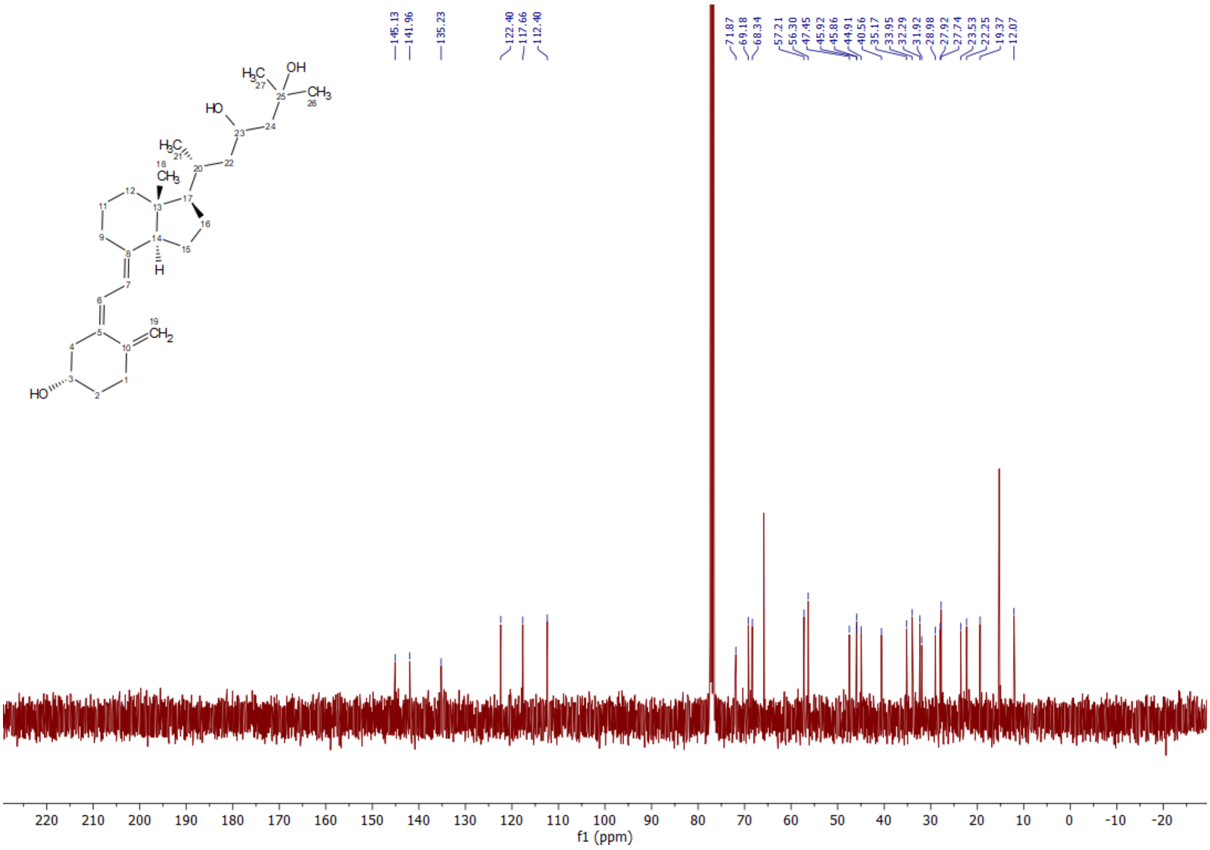

3 – ^{1}H NMR (500 MHz, CD_3OD)

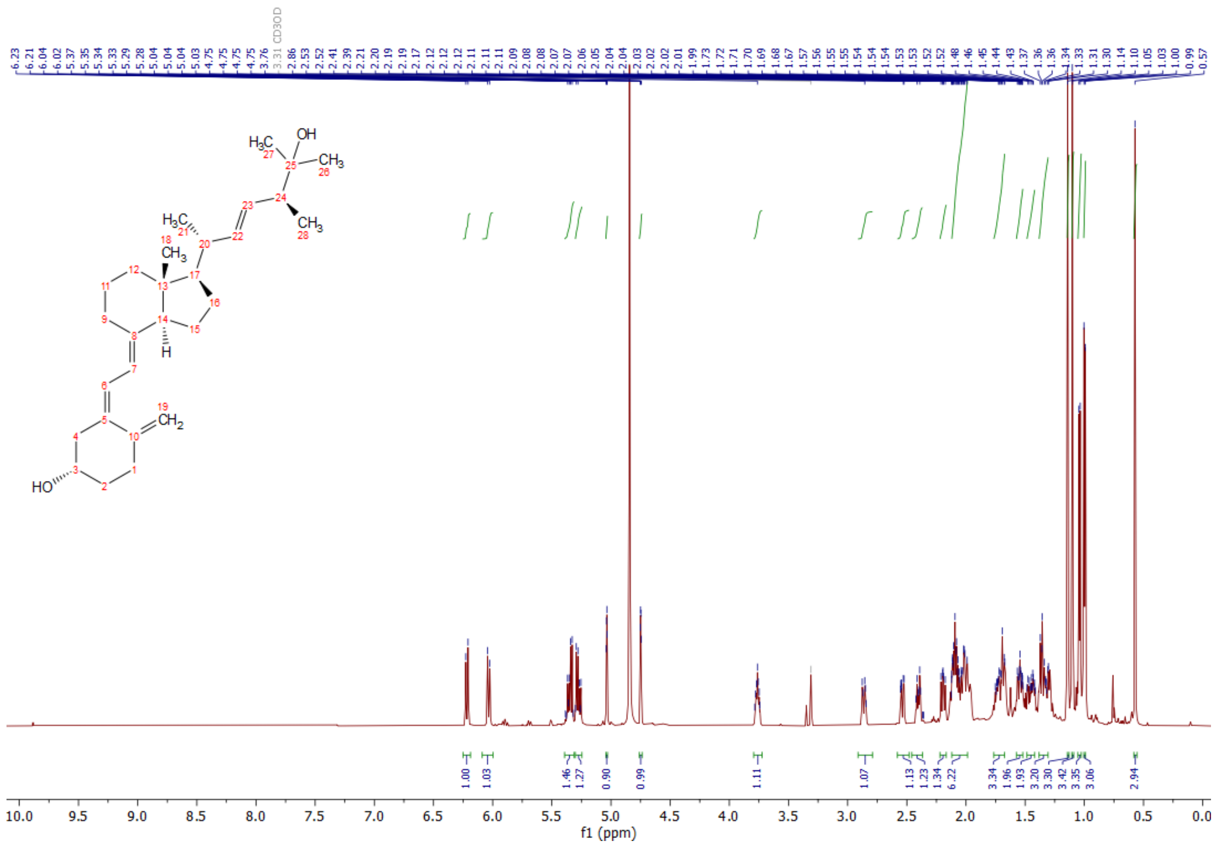

3 – ^{13}C NMR (126 MHz, CD_3OD)

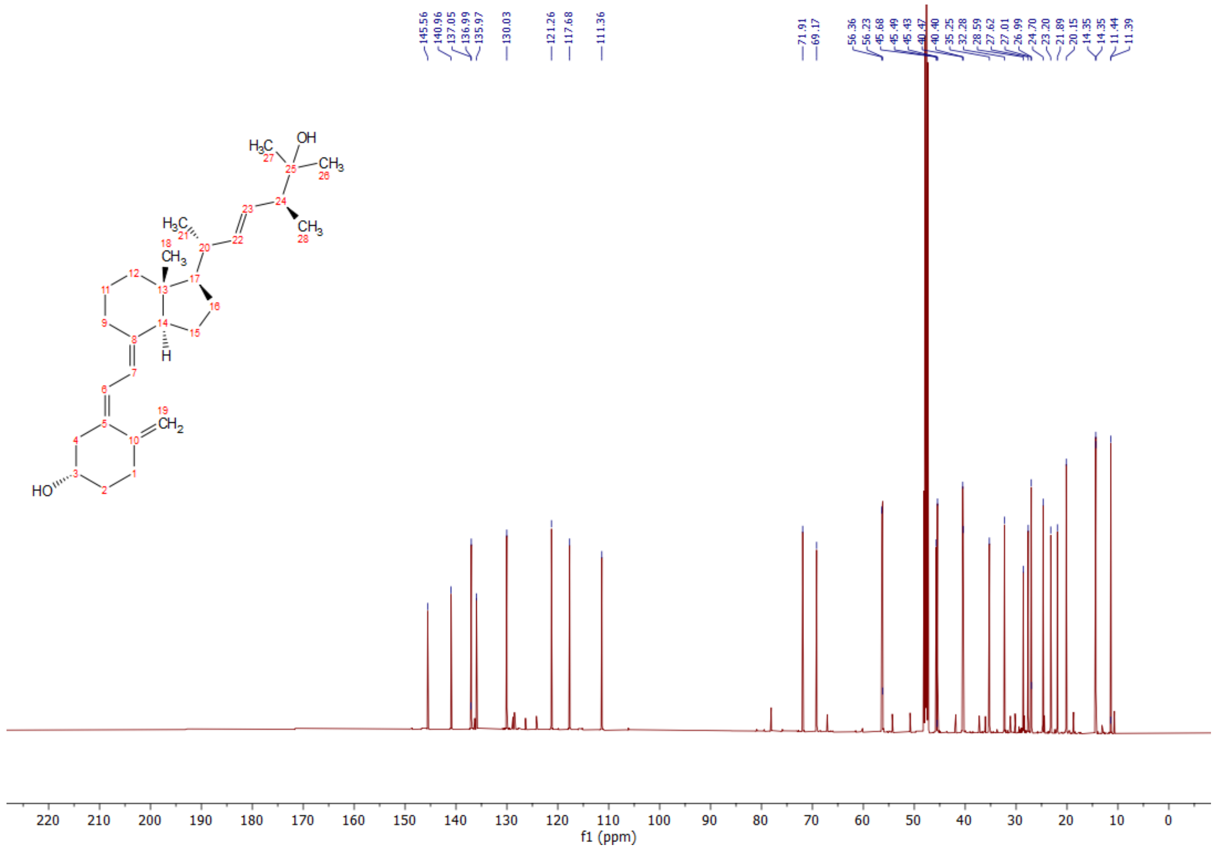

4 – ^{1}H NMR (400 MHz, CD_3OD)

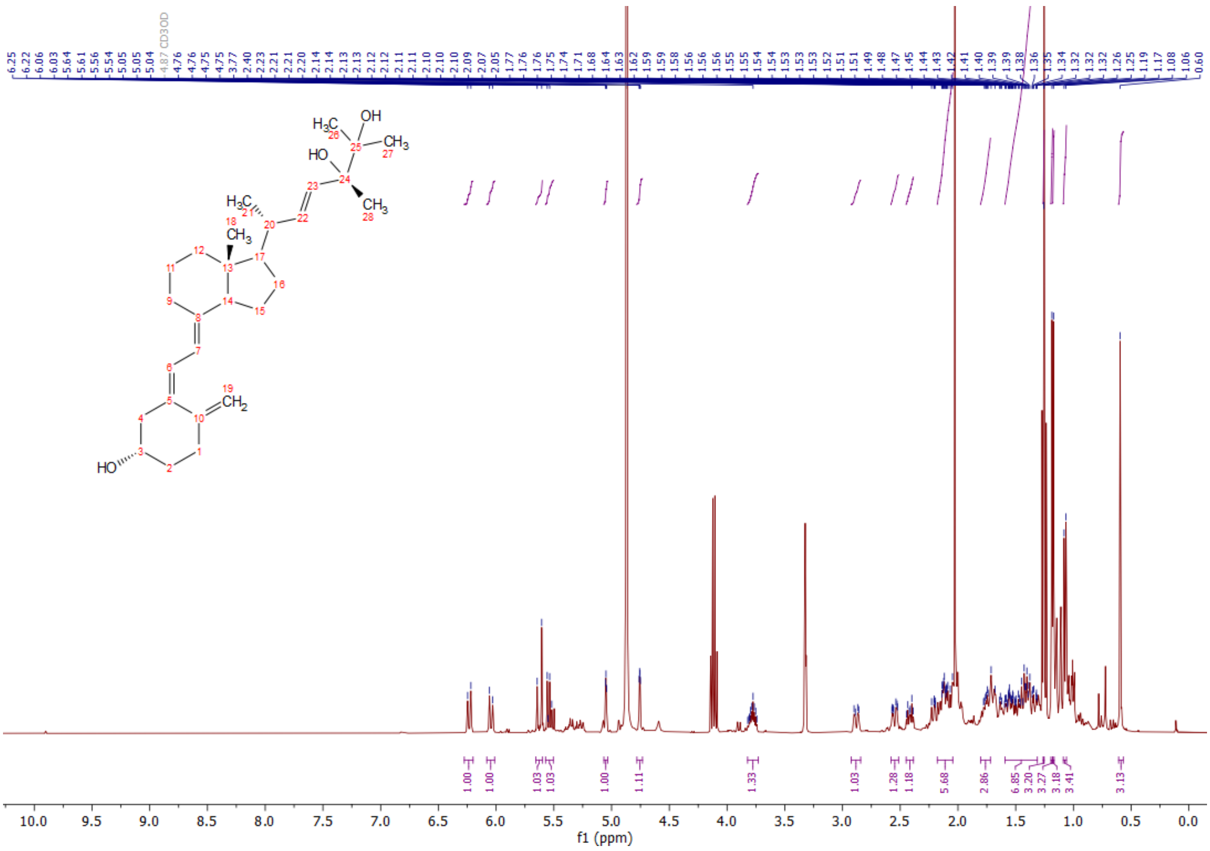

4 – ^{13}C NMR (101 MHz, CD_3OD)

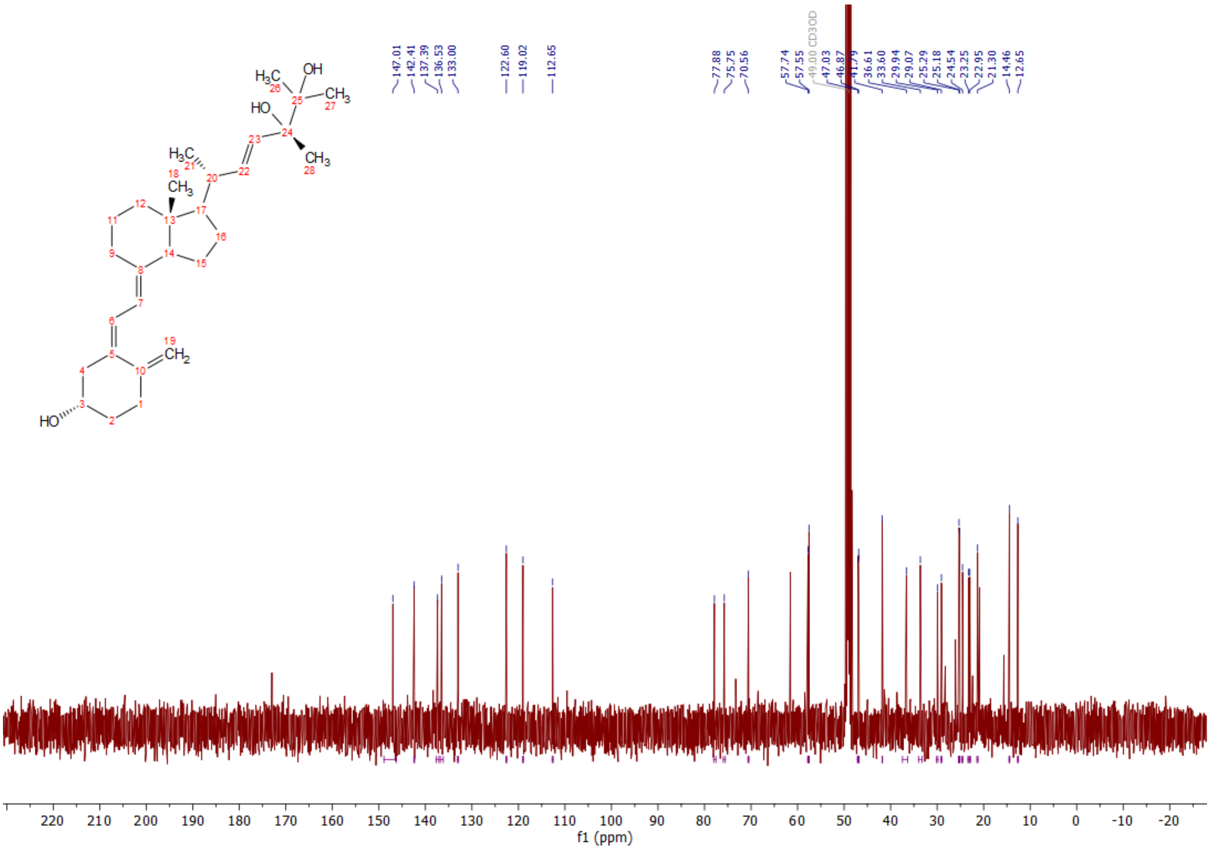

References

[33] C. J. C. Whitehouse, S. G. Bell, H. G. Tufton, R. J. Kenny, L. C. Ogilvie, L. L. Wong, *Chem. Commun.* **2008**, 966-968.

[34] a) C. J. C. Whitehouse, W. Yang, J. A. Yorke, B. C. Rowlatt, A. J. Strong, C. F. Blanford, S. G. Bell, M. Bartlam, L. L. Wong, Z. Rao, *Chembiochem* **2010**, *11*, 2549-2556; b) C. J. C. Whitehouse, W. Yang, J. A. Yorke, H. G. Tufton, L. C. Ogilvie, S. G. Bell, W. Zhou, M. Bartlam, Z. Rao, L. L. Wong, *Dalton Trans* **2011**, *40*, 10383-10396; c) X. Ren, J. A. Yorke, E. Taylor, T. Zhang, W. Zhou, L. L. Wong, *Chem. Eur. J.* **2015**, *21*, 15039-15047.

[35] T. Omura, R. Sato, *J. Biol. Chem.* **1964**, *239*, 2379–2385.

[36] D. C. Haines, D. R. Tomchick, M. Machius, J. A. Peterson, *Biochemistry* **2001**, *40*, 13456–13465.

[37] a) M. J. Abraham, T. Murtola, R. Schulz, S. Páll, J. C. Smith, B. Hess, E. Lindahl, *SoftwareX* **2015**, *1-2*, 19–25; b) B. Hess, *J. Chem. Theory Comput.* **2008**, *4*, 435–447.

[38] K. Lindorff-Larsen, S. Piana, K. Palmo, P. Maragakis, J. L. Klepeis, R. O. Dror, D. E. Shaw, *Proteins* **2010**, *78*, 1950–1958.

[39] W. L. Jorgensen, J. Chandrasekhar, J. D. Madura, R. W. Impey, M. L. Klein, *J. Chem. Phys.* **1983**, *79*, 926–935.

[40] K. Shahrokh, A. Orendt, G. S. Yost, T. E. Cheatham, 3rd, *J. Comput. Chem.* **2012**, *33*, 119–133.

[41] T. Darden, D. York, L. Pedersen, *J. Chem. Phys.* **1993**, *98*, 10089–10092.

[42] G. Bussi, D. Donadio, M. Parrinello, *J. Chem. Phys.* **2007**, *126*, 014101.

[43] H. J. C. Berendsen, J. P. M. Postma, W. F. van Gunsteren, A. DiNola, J. R. Haak, *J. Chem. Phys.* **1984**, *81*, 3684–3690.

[44] M. Parrinello, A. Rahman, *J. Appl. Phys.* **1981**, *52*, 7182–7190.

[45] W. H. Okamura, G. Zhu, D. K. Hill, R. J. Thomas, K. Ringe, D. B. Borchardt, A. W. Norman, L. J. Mueller, *J. Org. Chem.* **2002**, *67*, 1637-1650.

[46] Y. Tanaka, J. K. Wichmann, H. K. Schnoes, H. F. DeLuca, *Biochemistry* **1981**, *20*, 3875-3879.

[47] N. J. Koszewski, T. A. Reinhardt, D. C. Beitz, J. L. Napoli, E. G. Baggiolini, M. R. Uskokovic, R. L. Horst, *Analytical Biochemistry* **1987**, *162*, 446-452.

[48] K. Katsumi, T. Okano, Y. Ono, E. Maegaki, K. Nishimura, M. Baba, T. Kobayashi, O. Miyata, T. Naito, I. Ninomiya, *Chem. Pharm. Bull.* **1987**, *35*, 970–979.
